# Cancer-associated fibroblast derived CXCL14 drives cisplatin chemoresistance by enhancing nucleotide excision repair in bladder cancer

**DOI:** 10.1186/s13046-025-03487-4

**Published:** 2025-09-02

**Authors:** Tinghao Li, Kunyao Zhu, Hang Tong, Yan Sun, Junlong Zhu, Zijia Qin, Junrui Chen, Linfeng Wu, Xiaoyu Zhang, Aimin Wang, Xin Gou, Hubin Yin, Weiyang He

**Affiliations:** 1 Department of Urology, The First Affiliated Hospital of Chongqing Medical University, 1 Youyi Road, Yuzhong District, Chongqing, 400016 P.R. China; 2 Central Laboratory, The First Affiliated Hospital of Chongqing Medical University, Chongqing, 400016 China; 3 Chongqing Key Laboratory of Molecular Oncology and Epigenetics, The First Affiliated Hospital of Chongqing Medical University, Chongqing, 400016 China

**Keywords:** Cancer-associated fibroblast, Bladder cancer, Chemoresistance, DNA damage repair, CXCL14

## Abstract

**Background:**

A significant challenge in bladder cancer treatment is primary chemoresistance, in which cancer-associated fibroblasts (CAFs) in the tumor microenvironment (TME) play a pivotal role. While the contributions of CAFs to tumor progression and drug resistance are well established, the precise molecular mechanisms by which they induce chemoresistance remain unclear. A comprehensive understanding of the effect of TME modulation—particularly through CAFs—on the chemotherapeutic response is crucial for developing effective strategies to overcome chemoresistance and improve patient survival.

**Methods:**

Primary fibroblasts were isolated from paired clinical samples of bladder cancer tissues and adjacent normal tissues to identify key CAF-derived secretory factors. Bioinformatics analysis, semiquantitative RT‒qPCR, and dual-luciferase reporter assays were subsequently used to investigate the functional role and mechanistic basis of CXCL14 in chemoresistance. The therapeutic relevance of these findings was further evaluated through in vitro and in vivo models, including ex vivo patient-derived organoid (PDO) models, by assessing cisplatin sensitivity and validating therapeutic targeting of the CXCL14-CCR7-STAT3 axis with small molecule inhibitors.

**Results:**

Compared to normal fibroblasts and CAFs from nonchemoresistance groups, CAFs derived from cisplatin-resistant patients demonstrated significantly greater paracrine-mediated induction of chemoresistance. Mechanistically, CAF-secreted CXCL14 engaged CCR7 on bladder cancer cells, triggering STAT3 phosphorylation and consequently upregulating the DNA repair gene ERCC4 to promote cisplatin resistance. In vivo validation confirmed that pharmacological CCR7 or STAT3 inhibition markedly reversed chemoresistance and potentiated cisplatin-induced tumor cell death. Notably, STAT3 activation mediated the overexpression of the glycolytic enzymes HK2 and LDHA, resulting in greater glycolytic flux in resistant cells. This metabolic reprogramming further facilitated the transdifferentiation of normal fibroblasts into CXCL14-secreting CAFs, establishing a self-reinforcing feedback loop that sustains chemoresistance.

**Conclusion:**

The CXCL14/CCR7/STAT3 axis critically mediates cisplatin resistance in bladder cancer through dual modulation of DNA repair and glycolytic metabolism. Therapeutic cotargeting of this pathway with CCR7 or STAT3 inhibitors combined with cisplatin represents a promising strategy to overcome chemoresistance and improve clinical outcomes.

**Supplementary Information:**

The online version contains supplementary material available at 10.1186/s13046-025-03487-4.

## Introduction

Bladder cancer is the predominant type of urothelial carcinoma and accounts for 3% of global cancer mortality [1]. Primary chemoresistance poses a major barrier in patients ineligible for surgery, for whom platinum-based chemotherapy remains the standard regimen for advanced or metastatic bladder cancer [2]. While platinum agents exert cytotoxicity by inducing DNA double-strand breaks (DSBs) and activating the DNA damage response (DDR), intrinsic resistance mechanisms frequently compromise therapeutic efficacy [3]. Critically, nearly 50% of muscle-invasive bladder cancer (MIBC) patients are reportedly cisplatin-ineligible due to preexisting resistance or comorbidities [4–6]. This high prevalence of primary chemoresistance leads to DDR pathway hyperactivation, attenuated cancer cell death, and ultimately poor prognosis. It is therefore urgent to overcome this limitation to improve cisplatin response rates and survival outcomes.

Multiple factors may contribute to DNA damage repair, rather than focusing on the development of chemoresistance within cancer cells themselves, this study focuses on alterations in the tumor microenvironment (TME) that play crucial roles in regulating the malignant progression of cancer cells [7]. The TME is known as a multicellular system comprising various mesenchymal and immune cells. Among the major cellular components of the tumor stroma, the cross talk between cancer associated fibroblasts (CAFs) and tumor cells holds great value in the development of chemoresistance in most solid cancers [8]. Transcriptionally and functionally heterogeneous subsets of CAFs have been identified in several solid cancers, and different CAFs have been reported to promote the chemoresistance of cancer cells by not only remodeling the extracellular matrix and forming a barrier for drug or therapeutic immune cell penetration, but also secreting various cytokines or metabolites and affecting targeted tumor cells [9–11]. While growing evidence underscores the intricate interplay between CAFs and cancer cells, which significantly impacts their sensitivity to anticancer therapeutics [12], how CAFs regulate the development of chemoresistance in bladder cancer cells is not as well explored.

CAFs often secrete chemokines that significantly impact cancer cell behavior, with both CXC and CC chemokines playing crucial roles in regulating DDR mechanisms [13]. For example, Dedobbeleer et al. demonstrated that stromal-derived C-X-C motif chemokine ligand 12 (CXCL12) reduces cell death in glioblastoma and enhances DNA repair by modulating key apoptotic regulators and stabilizing the DNA repair protein RAD51 [14]. CXCL14, also known as breast and kidney-expressed chemokine (BRAK), has traditionally been considered an inflammatory and immune modulator. However, its effects can vary across different tumors depending on the cellular context [15]. Recent studies suggest that CXCL14 derived from CAFs may promote malignant progression in ovarian and breast cancers [16, 17]. Nevertheless, the role of CXCL14 in bladder cancer remains to be fully elucidated.

In the present study, we focused on the role of CAFs in facilitating chemoresistance in bladder cancer cells. For the first time, we identify the crucial role of CAF-derived CXCL14 in regulating the DDR and glucose metabolic reprogrammig of cancer cells. Additionally, the combination of cisplatin-based chemotherapy with CCR7 or STAT3-targeted therapies may offer a more effective treatment strategy for patients with bladder cancer.

## Materials and methods

### Clinical tissues collection and primary fibroblasts isolation

Bladder cancer tissues and adjacent normal tissues were collected from patients undergoing radical cystectomy for bladder cancer at The First Affiliated Hospital of Chongqing Medical University. All samples were confirmed to originate from urothelial tissue, as verified by radiomic analysis before surgery and pathological examination after surgery. Adjacent tissues were collected from those located more than 5 cm away from the cancer tissues. Patients were stratified into two groups: the naïve group and the chemotherapy-treated group based on their receipt of platinum-based chemotherapy prior to surgery. Subsequently, the chemotherapy-treated group was further evaluated based on their response to chemotherapeutic agents, monitored by contrast-enhanced CT scans conducted before and after treatment: patients exhibiting progressive disease (PD) or stable disease (SD) were categorized as chemoresistant bladder cancer patients, while those demonstrating complete or partial response were classified as chemosensitive.

For primary fibroblast isolation, fresh cancer and adjacent tissues were obtained from patients undergoing radical cystectomy, and transferred on ice. After eliminating blood, connective tissue and vessels, tissues were digested for 1 h at 37 °C with 1 mg/ml collagenase A (Solarbio, China, cat. C8140). Enzymatic digestion was halted and then filtered through 70 µm cell strainers and centrifuged. Cells were seeded undisturbed in DMEM/F12 containing 10% fetal bovine serum (FBS) for 3 days at 37 °C with 5% CO_2_. Nonadherent cells were discarded, and the remaining cells underwent differential trypsinization. Following centrifugation and cell counting, cell pellet was resuspended and mixed with anti-fibroblast microbeads (Miltenyi Biotec, Germany, cat. 130–050-601). The mixture was resuspended in 500 µL buffer. The cell suspension was applied onto the column, which was placed in the magnetic field of a suitable+ MACS separator (Miltenyi Biotec, cat. 130–042-501), and the flow-through containing unlabeled cells was discarded. After removing the separator, moderate buffer was dripped and the magnetically labeled cells were flushed out. The isolated primary fibroblasts were then incubated for further experiments.

Collection of clinical specimens and related procedures were conducted with approval from the Internal Review and Ethics Committee of The First Affiliated Hospital of Chongqing Medical University, and all patients provided written informed consent.

### Cell lines and culture

Two strains of human bladder cancer cells (T24 and UM-UC-3) were purchased from the Cell Bank of the Chinese Academy of Sciences, Shanghai. All cells were confirmed to have undergone appropriate STR testing at the time of purchase, and mycoplasma contamination was excluded. All cell lines were maintained in a 5% CO_2_ incubator at 37 °C. T24 cells were cultured in Roswell Park Memorial Institute (RPMI) 1640 medium, while UM-UC-3 cells were cultured in DMEM, and supplemented with 10% FBS (Gibco, USA). Various inhibitors including doxycycline (200 ng/mL; GLPBIO, USA, cat. GC13750), STAT3 inhibitor (STAT3i) (S3I-201,5 µM; Selleck, USA, cat. S1155), and CCR7 inhibitor (CCR7i) (Cmp2105, 50 µM; MCE, USA, cat. HY-133073) were employed to treat the cell lines as specified. In experiments using antibody neutralization, cells were pre-incubated with CXCL14 or IgG antibodies for 1 h prior to subsequent experiments.

### Immunohistochemical (IHC) staining

Tissue slides were obtained from The First Affiliated Hospital of Chongqing Medical University. All samples were fixed in formalin and embedded in paraffin. Following dewaxing with dimethylbenzene and hydration in gradient ethanol, sections underwent a 15 min incubation in 3% H_2_O_2_ at room temperature. Subsequently, antigen retrieval was achieved by incubating the sections in Tris–EDTA buffer (pH 9.0) at 100 °C for 30 min. After blocking, the sections were subsequently incubated overnight at 4 °C with primary antibodies (anti-alpha smooth muscle actin (α-SMA), Abcam, cat. ab7817; anti-cleaved caspase 3, Proteintech, cat. 19,677–1-AP; anti-Ki67, Proteintech, cat. 27,309–1-AP). After that, the sections were incubated with secondary antibodies and then stained with 3,3'-Diaminobenzidine (DAB) and hematoxylin. Positive cells were characterized by distinct brown staining. Images of the sections were captured under a microscope in five random fields.

### Mouse xenograft assays

Four to six-week-old BALB/c nude mice (Vital River, China) were fed under standard conditions. Subsequently, they were subcutaneously injected with either 2 × 10^6 T24 cells alone or co-injected with 1 × 10^5 CAFs or NFs with different pretreatment. Once the average tumor volume reached approximately 200 mm^3, the tumor-bearing mice were administered cisplatin (3 mg/kg) intraperitoneally. This treatment was given in combination with CCR7i (7.3 µg/kg) or STAT3i (10 mg/kg), or an equivalent volume of PBS. All treatments were administered twice a week. Tumor size was assessed every 3–5 days using a caliper, and tumor volume was calculated using the formula: tumor volume (mm^3) = 0.5 × longest diameter × shortest diameter^2. At the end of each experiment, all nude mice were euthanized, and their tumors were carefully dissected and processed for immunohistochemical analyses. All animal procedures were conducted in accordance with the guidelines of the Institutional Animal Care and Use Committee (IACUC) of Chongqing Medical University and adhered to the ARRIVE guidelines.

### Cells transfection

Plasmids carrying short hairpin RNA (shRNA) targeting human CXCL14, ERCC4, MRP2 and CCR7 were constructed and obtained from Tsingke (Beijing, China). Scrambled control shRNAs for each gene were utilized as control. The pLKO.1-mCherry-puro vector was used to clone shRNAs. Stable expression of shRNA was obtained by lentiviral transduction of CAFs, T24 and UM-UC-3 cells. Lentivirus was packaged by co-transfected target plasmids having the psPAX2/pMD2.G in 293 T cells using a Lipo3000 transfection reagent according to the manufacturer's instructions (GLPBIO, USA, cat. GK20006). In brief, total of 1 × 10^5 CAFs, UM-UC-3 or T24 cells were transfected with 2 × 10^6 virus (multiplicity of infection = 20) for 48 h. Then, the cells were transferred to fresh medium containing puromycin (2 ug/ml) for the selection of stable clones. The transfection efficiency was assessed by FL-2 channel detection of fluorescence generated from mCherry-labeled shRNAs. The shRNA sequences are listed in Table 1.

**Table 1:** Sequences of shRNA or primer sequences for RT-qPCR

Item	Genes	Sequences
Primer	MRP2	forward 5’-CCCTGCTGTTCGATATACCAATC-3'
		reverse 5’-TCGAGAGAATCCAGAATAGGGAC-3’
	ATP7B	forward 5’-ATATTGAGCGGTTACAAAGCACT-3'
		reverse 5’-TGCCCCAAGGTCTCAGAATTA-3’
	CTR1	forward 5’-GGGGATGAGCTATATGGACTCC-3'
		reverse 5’-TCACCAAACCGGAAAACAGTAG-3’
	XIAP	forward 5’-AATAGTGCCACGCAGTCTACA-3'
		reverse 5’-CAGATGGCCTGTCTAAGGCAA-3’
	ERCC1	forward 5’-CTACGCCGAATATGCCATCTC-3'
		reverse 5’-GTACGGGATTGCCCCTCTG-3’
	ERCC4	forward 5’-CCTCTTTCGCCAGAAAAACAAAC-3'
		reverse 5’-TTTACTGCTACATGGAACCTTGG-3’
	GSTK1	forward 5’-TCTGGAAAAGATCGCAACGC-3'
		reverse 5’-GCCCAAAGGCTCCGTATCTG-3’
	ATP7B	forward 5’-ATATTGAGCGGTTACAAAGCACT-3'
		reverse 5’-TGCCCCAAGGTCTCAGAATTA-3’
	IL1A	forward 5’-TGTATGTGACTGCCCAAGATGAA-3'
		reverse 5’-GGATGGGCAACTGATGTGAAATA-3’
	CCL2	forward 5’-GGCTGAGACTAACCCAGAAACAT-3'
		reverse 5’-ACTTGCTGCTGGTGATTCTTCTA-3’
	CCL7	forward 5’-TACTTCAACTACCTGCTGCTACA-3'
		reverse 5’-GCACAGATCTCCTTGTCCAGTTT-3’
	CLCF1	forward 5’-TTGGCTGGGACCTATCTGAACTA-3'
		reverse 5’-GTCAGCCGCAGTTTGTCATTGA-3’
	CSF3	forward 5’-GCTGCTCGGACACTCTCTG-3'
		reverse 5’-GCCATTCCCAGTTCTTCCATCTG-3’
	CXCL14	forward 5’-ACTGCGAGGAGAAGATGGTTATC-3'
		reverse 5’-CAGGCGTTGTACCACTTGATGAA-3’
	DKK2	forward 5’- GCCAAACTCAACTCCATCAAGTC −3'
		reverse 5’- CCTCCCAACTTCACACTCCTTAT −3’
	EREG	forward 5’- ATCACAGTCGTCGGTTCCACATA −3'
		reverse 5’- TGACCTAACACTTGACCCAACAT −3’
	GDNF	forward 5’- GCAGTGACTCAAATATGCCAGAG −3'
		reverse 5’- TCTTCTAGGAAGCACTGCCATTT-3’
	IGFBP5	Forward 5’-GTGAAGAAGGACCGCAGAAAGAA-3'
		reverse 5’-AGAATCCTTTGCGGTCACAATTG-3’
	IL11	forward 5’-CCTGAAGACCCTGGAGCCC-3'
		reverse 5’-CACAGCCGAGTCTTCAGCA-3’
	IL6	forward 5’-AACATGTGTGAAAGCAGCAAAGA-3'
		reverse 5’-CTCTGGCTTGTTCCTCACTACTC-3’
	LIF	forward 5’-CAAACCAAGTCAGGCTCCAGTAT-3'
		reverse 5’-CTGCCGCCAAGACCTTCATTAT-3’
	NGF	forward 5’-TACCAAGGGAGCAGCTTTCTATC-3'
		reverse 5’-AGTGTCAAGGGAATGCTGAAGTT-3’
	VGF	forward 5’-GACCCTCCTCTCCACCTCTC-3'
		reverse 5’-ACCGGCTCTTTATGCTCAGA-3’
	ACTB	forward 5’-AGAAAATCTGGCACCACACCT-3'
		reverse 5’-GATAGACAGCCTGGATAGCA-3’
	STAT3	forward 5’-GGTGCCTGTGGGAAGAATCA-3'
		reverse 5’-ACATCCTGAAGGTGCTGCTC-3’
	CCR7	forward 5’-TCTGCCTGGACTAGAGGGAC-3'
		reverse 5’-TATCTTCTGGAGCAGGGGCT-3’
	CXCR4	forward 5’-CAGCGTCTCAGTGCCCTTT-3'
		reverse 5’-CTTGGCCTCTGACTGTTGGT-3’
	ACKR3	forward 5’-GGTTGTCCTCACCATCCCAG-3'
		reverse 5’-AGAAGATGTCCAGCAGCACC-3’
	ACKR2	forward 5’-GGACTGGGCATTTCCTTCCA-3'
		reverse 5’-TTGCAGGTCCAACAGCGTAT −3’
	G6PD	forward 5’-AACATCGCCTGCGTTATCC-3'
		reverse 5’-TGACCTTCTCATCACGGACG-3’
	HK2	forward 5’-GCCCGCCAGAAGACATTAG-3'
		reverse 5’-TGCTCAGACCTCGCTCCAT −3’
	PDK1	forward 5’-GAGGGTTACGGGACAGATGC-3'
		reverse 5’-GCCTCGTGGTTGGTGTTGT-3’
	PKM	forward 5’-GCTGTGGACTTGCCTGCTGT-3'
		reverse 5’-GCCTTGCGGATGAATGACG-3’
	GLUT1	forward 5’-GTATGTGGAGCAACTGTGTGGT-3'
		reverse 5’-CTCGGGTGTCTTGTCACTTTG-3’
	LDHA	forward 5’-ATTAAGCTGTCATGGGTGGGTC-3'
		reverse 5’-CAGAGAGACACCAGCAACATTCA-3’
	PFKP	forward 5’-TGTATTCAGAAGAGGGCAAAGG-3'
		reverse 5’-AGTTTCTATCAAATGGAGAGGGTG-3’
Target Sequences of shRNA	shCXCL14-1	5’-GCGCTTCATCAAGTGGTACAA-3'
	shCXCL14-2	5’-CGACGTGAAGAAGCTGGAAAT-3'
	shERCC4-1	5’-ACAAGACAATCCGCCATTA-3’
	shERCC4-2	5’-GGTTATTCAGAAGGTACAAGG-3’
	shMRP2-1	5’-GGAGTACACCGTTGGAGAAAC-3’
	shMRP2-2	5’-GCTGTGGTCAAGTGTTCTACA-3’
	shCCR7	5’-GATGAGGTCACGGACGATT-3’
	shCXCR4	5’-CATCATCTTCTTAACTGGCAT-3’
	shACKR2	5’-CGGGAGTTTCTTGTGCAAGAT-3’
	shACKR3	5’-CTTCTCCATTATCGCTGTCTT-3’

All primers and shRNAs were designed to target Homo sapiens

### Constitutive and Tetracycline regulated (Tet-On) lentiviral expression system

Lentiviral expression system of shCCR7 were designed and synthesized by Tsingke (Beijing, China). In briefly, human CCR7-shRNA were constructed and inserted into the lentiviral vector pLV34ltr-hPGK-TetR-IRES-Puro (Table 2). Then, obtained stably transfected cells were selected by treatment with 1 µg/mL of puromycin after 200 ng/mL of doxycycline pretreatment. The transfection efficiency of shCCR7 on T24 cells was determined using western blotting.

**Table 2:** Gene sequence used in luciferase reporter assay

Gene	Sequence (5’−3’)
ERCC4-WT	ATGGGGAGTAATCGCAAAAGGAAGGGCCAAAAACCACCCTAGGAAACAGGTGCTACTAACTCTTGGCGCATTTGGAATATTGACCAGTAACTTTTTCAACTGTCGATGTCGGGGGATTAAGCTCCTTAGGCTTACTTCCCCTTCCCTTGCTTTTTGGCAGCTTGAGGCTAGGGTGCCAGGGGATGTGGAAACTCAAAAGAGACGTGAATGGAGCCTGATATCTGCAAGCTACACCGACAGGGGGCGCCAGCCCCTACAGGTGACTCCATGAATCTTCGGCTCCACTCGGCTCTTCTTCGGCTGAGTTCGGCCAACGCTTGCCTTCTCAGGCTCGGCTCTCTTCGGCTTTCTGAGAGCGCGCCTGTCGTGGCCTCGGCTCCTCTTCGGCTGCGTTCGGCCCACGATCATCTCAGTCTCAGCTCTCCTCGGCTACGTTCGGCTGGCTGCCGTCCTCTCGGACTCGGCTCTCTTCGGTTGAGTTCGGCCTACTCTCCACTAGGAGTCGGCTTC
ERCC4-mut-1	ATGGGGAGTAATCGCAAAAGGAAGGGCCAAAAACCACCCTAGGAAACAGGTGCTACTAACTCTTGGCGCATTTGGAATATTGACCAGTAACTTTTTCAACTGTCGATGTCGGGGGATTAAGCTCCTTAGGCTTACTTCCCCTTCCCTTGAGGGGGTTACTCTTGAGGCTAGGGTGCCAGGGGATGTGGAAACTCAAAAGAGACGTGAATGGAGCCTGATATCTGCAAGCTACACCGACAGGGGGCGCCAGCCCCTACAGGTGACTCCATGAATCTTCGGCTCCACTCGGCTCTTCTTCGGCTGAGTTCGGCCAACGCTTGCCTTCTCAGGCTCGGCTCTCTTCGGCTTTCTGAGAGCGCGCCTGTCGTGGCCTCGGCTCCTCTTCGGCTGCGTTCGGCCCACGATCATCTCAGTCTCAGCTCTCCTCGGCTACGTTCGGCTGGCTGCCGTCCTCTCGGACTCGGCTCTCTTCGGTTGAGTTCGGCCTACTCTCCACTAGGAGTCGGCTTC
ERCC4-mut-2	ATGGGGAGTAATCGCAAAAGGAAGGGCCAAAAACCACCCTAGGAAACAGGTGCTACTAACTCTTGGCGCATTTGGAATATTGACCAGTAACTTTTTCAACTGTCGATGTCGGGGGATTAAGCTCCTTAGGCTTACTTCCCCTTCCCTTGCTTTTTGGCAGCTTGAGGCTAGGGTGCCAGGGGATGTGGAAACTCAAAAGAGACGTGAATGGAGCCTGATATCTGCAAGCTACACCGACAGGGGGCGCCAGCCCCTACAGGTGACTCCATGAATCTTCGGCTCCACTCGGCTCTTCTTCGGCTGAGTTCGGCCAACGCTTGCCTTCTCAGGCTCGGCTCTCTTCGGCGGGAGTCTCTAGCGCCTGTCGTGGCCTCGGCTCCTCTTCGGCTGCGTTCGGCCCACGATCATCTCAGTCTCAGCTCTCCTCGGCTACGTTCGGCTGGCTGCCGTCCTCTCGGACTCGGCTCTCTTCGGTTGAGTTCGGCCTACTCTCCACTAGGAGTCGGCTTC
ERCC4-mut-1 + 2	ATGGGGAGTAATCGCAAAAGGAAGGGCCAAAAACCACCCTAGGAAACAGGTGCTACTAACTCTTGGCGCATTTGGAATATTGACCAGTAACTTTTTCAACTGTCGATGTCGGGGGATTAAGCTCCTTAGGCTTACTTCCCCTTCCCTTGAGGGGGTTACTCTTGAGGCTAGGGTGCCAGGGGATGTGGAAACTCAAAAGAGACGTGAATGGAGCCTGATATCTGCAAGCTACACCGACAGGGGGCGCCAGCCCCTACAGGTGACTCCATGAATCTTCGGCTCCACTCGGCTCTTCTTCGGCTGAGTTCGGCCAACGCTTGCCTTCTCAGGCTCGGCTCTCTTCGGCGGGAGTCTCTAGCGCCTGTCGTGGCCTCGGCTCCTCTTCGGCTGCGTTCGGCCCACGATCATCTCAGTCTCAGCTCTCCTCGGCTACGTTCGGCTGGCTGCCGTCCTCTCGGACTCGGCTCTCTTCGGTTGAGTTCGGCCTACTCTCCACTAGGAGTCGGCTTC
STAT3	ATGGCCCAATGGAATCAGCTACAGCAGCTTGACACACGGTACCTGGAGCAGCTCCATCAGCTCTACAGTGACAGCTTCCCAATGGAGCTGCGGCAGTTTCTGGCCCCTTGGATTGAGAGTCAAGATTGGGCATATGCGGCCAGCAAAGAATCACATGCCACTTTGGTGTTTCATAATCTCCTGGGAGAGATTGACCAGCAGTATAGCCGCTTCCTGCAAGAGTCGAATGTTCTCTATCAGCACAATCTACGAAGAATCAAGCAGTTTCTTCAGAGCAGGTATCTTGAGAAGCCAATGGAGATTGCCCGGATTGTGGCCCGGTGCCTGTGGGAAGAATCACGCCTTCTACAGACTGCAGCCACTGCGGCCCAGCAAGGGGGCCAGGCCAACCACCCCACAGCAGCCGTGGTGACGGAGAAGCAGCAGATGCTGGAGCAGCACCTTCAGGATGTCCGGAAGAGAGTGCAGGATCTAGAACAGAAAATGAAAGTGGTAGAGAATCTCCAGGATGACTTTGATTTCAACTATAAAACCCTCAAGAGTCAAGGAGACATGCAAGATCTGAATGGAAACAACCAGTCAGTGACCAGGCAGAAGATGCAGCAGCTGGAACAGATGCTCACTGCGCTGGACCAGATGCGGAGAAGCATCGTGAGTGAGCTGGCGGGGCTTTTGTCAGCGATGGAGTACGTGCAGAAAACTCTCACGGACGAGGAGCTGGCTGACTGGAAGAGGCGGCAACAGATTGCCTGCATTGGAGGCCCGCCCAACATCTGCCTAGATCGGCTAGAAAACTGGATAACGTCATTAGCAGAATCTCAACTTCAGACCCGTCAACAAATTAAGAAACTGGAGGAGTTGCAGCAAAAAGTTTCCTACAAAGGGGACCCCATTGTACAGCACCGGCCGATGCTGGAGGAGAGAATCGTGGAGCTGTTTAGAAACTTAATGAAAAGTGCCTTTGTGGTGGAGCGGCAGCCCTGCATGCCCATGCATCCTGACCGGCCCCTCGTCATCAAGACCGGCGTCCAGTTCACTACTAAAGTCAGGTTGCTGGTCAAATTCCCTGAGTTGAATTATCAGCTTAAAATTAAAGTGTGCATTGACAAAGACTCTGGGGACGTTGCAGCTCTCAGAGGATCCCGGAAATTTAACATTCTGGGCACAAACACAAAAGTGATGAACATGGAAGAATCCAACAACGGCAGCCTCTCTGCAGAATTCAAACACTTGACCCTGAGGGAGCAGAGATGTGGGAATGGGGGCCGAGCCAATTGTGATGCTTCCCTGATTGTGACTGAGGAGCTGCACCTGATCACCTTTGAGACCGAGGTGTATCACCAAGGCCTCAAGATTGACCTAGAGACCCACTCCTTGCCAGTTGTGGTGATCTCCAACATCTGTCAGATGCCAAATGCCTGGGCGTCCATCCTGTGGTACAACATGCTGACCAACAATCCCAAGAATGTAAACTTTTTTACCAAGCCCCCAATTGGAACCTGGGATCAAGTGGCCGAGGTCCTGAGCTGGCAGTTCTCCTCCACCACCAAGCGAGGACTGAGCATCGAGCAGCTGACTACACTGGCAGAGAAACTCTTGGGACCTGGTGTGAATTATTCAGGGTGTCAGATCACATGGGCTAAATTTTGCAAAGAAAACATGGCTGGCAAGGGCTTCTCCTTCTGGGTCTGGCTGGACAATATCATTGACCTTGTGAAAAAGTACATCCTGGCCCTTTGGAACGAAGGGTACATCATGGGCTTTATCAGTAAGGAGCGGGAGCGGGCCATCTTGAGCACTAAGCCTCCAGGCACCTTCCTGCTAAGATTCAGTGAAAGCAGCAAAGAAGGAGGCGTCACTTTCACTTGGGTGGAGAAGGACATCAGCGGTAAGACCCAGATCCAGTCCGTGGAACCATACACAAAGCAGCAGCTGAACAACATGTCATTTGCTGAAATCATCATGGGCTATAAGATCATGGATGCTACCAATATCCTGGTGTCTCCACTGGTCTATCTCTATCCTGACATTCCCAAGGAGGAGGCATTCGGAAAGTATTGTCGGCCAGAGAGCCAGGAGCATCCTGAAGCTGACCCAGGTAGCGCTGCCCCATACCTGAAGACCAAGTTTATCTGTGTGACACCAACGACCTGCAGCAATACCATTGACCTGCCGATGTCCCCCCGCACTTTAGATTCATTGATGCAGTTTGGAAATAATGGTGAAGGTGCTGAACCCTCAGCAGGAGGGCAGTTTGAGTCCCTCACCTTTGACATGGAGTTGACCTCGGAGTGCGCTACCTCCCCCATGTG
ERCC4	ATGGAGTCAGGGCAGCCGGCTCGACGGATTGCCATGGCGCCGCTGCTGGAGTACGAGCGACAGCTGGTGCTGGAACTGCTCGACACTGACGGGCTAGTAGTGTGCGCCCGCGGGCTCGGCGCGGACCGGCTCCTCTACCACTTTCTCCAGCTGCACTGCCACCCAGCCTGCCTGGTGCTGGTGCTCAACACGCAGCCGGCCGAGGAGGAGTATTTTATCAATCAGCTGAAGATAGAAGGAGTTGAACACCTCCCTCGCCGTGTAACAAATGAAATCACAAGCAACAGTCGCTATGAAGTTTACACACAAGGTGGTGTTATATTTGCGACAAGTAGGATACTTGTGGTTGACTTCTTGACTGATAGAATACCTTCAGATTTAATTACTGGCATCTTGGTGTATAGAGCCCACAGAATAATCGAGTCTTGTCAAGAAGCATTCATCTTGCGCCTCTTTCGCCAGAAAAACAAACGTGGTTTTATTAAAGCTTTCACAGACAATGCTGTTGCCTTTGATACTGGTTTTTGTCATGTGGAAAGAGTGATGAGAAATCTTTTTGTGAGGAAACTGTATCTGTGGCCAAGGTTCCATGTAGCAGTAAACTCATTTTTAGAACAGCACAAACCTGAAGTTGTAGAAATCCATGTTTCTATGACACCTACCATGCTTGCTATACAGACTGCTATACTGGACATTTTAAATGCATGTCTAAAGGAACTAAAATGCCATAACCCATCGCTTGAAGTGGAAGATTTATCTTTAGAAAATGCTATTGGAAAACCTTTTGACAAGACAATCCGCCATTATCTGGATCCTTTGTGGCACCAGCTTGGAGCCAAGACTAAATCCTTAGTTCAGGATTTGAAGATATTACGAACTTTGCTGCAGTATCTCTCTCAGTATGATTGTGTCACATTTCTTAATCTTCTGGAATCTCTGAGAGCAACGGAAAAAGCTTTTGGTCAGAATTCAGGTTGGCTGTTTCTTGACTCCAGCACCTCGATGTTTATAAATGCTCGAGCAAGGGTTTATCATCTTCCAGATGCCAAAATGAGTAAAAAAGAAAAAATATCTGAAAAAATGGAAATTAAAGAAGGGGAAGAAACAAAAAAGGAACTGGTCCTAGAAAGCAACCCAAAGTGGGAGGCACTGACTGAAGTATTAAAAGAAATTGAGGCAGAAAATAAGGAGAGTGAAGCTCTTGGTGGTCCAGGTCAAGTACTGATTTGTGCAAGTGATGACCGAACATGTTCCCAGCTGAGAGACTATATCACTCTTGGAGCGGAGGCCTTCTTATTGAGGCTCTACAGGAAAACCTTTGAGAAGGATAGCAAAGCTGAAGAAGTCTGGATGAAATTTAGGAAGGAAGACAGTTCAAAGAGAATTAGGAAATCTCACAAAAGACCTAAAGACCCCCAAAACAAAGAACGGGCTTCTACCAAAGAAAGAACCCTCAAAAAGAAAAAACGGAAGTTGACCTTAACTCAAATGGTAGGAAAACCTGAAGAACTGGAAGAGGAAGGAGATGTCGAGGAAGGATATCGTCGAGAAATAAGCAGTAGCCCAGAAAGCTGCCCGGAAGAAATTAAGCATGAAGAATTTGATGTAAATTTGTCATCGGATGCTGCTTTCGGAATCCTGAAAGAACCCCTCACTATCATCCATCCGCTTCTGGGTTGCAGCGACCCCTATGCTCTGACAAGGGTACTACATGAAGTGGAGCCAAGATACGTGGTTCTTTATGACGCAGAGCTAACCTTTGTTCGGCAGCTTGAAATTTACAGGGCGAGTAGGCCTGGGAAACCTCTGAGGGTTTACTTTCTTATATACGGAGGTTCAACTGAGGAACAACGCTATCTCACTGCTTTGCGGAAAGAAAAGGAAGCTTTTGAAAAACTCATAAGGGAAAAAGCAAGCATGGTTGTCCCTGAAGAAAGAGAAGGCAGAGATGAAACAAACTTAGACCTAGTAAGAGGCACAGCATCTGCAGATGTTTCCACTGACACTCGGAAAGCCGGTGGCCAGGAACAGAATGGTACACAGCAAAGCATAGTTGTGGATATGCGTGAATTTCGAAGTGAGCTTCCATCTCTGATCCATCGTCGGGGCATTGACATTGAACCCGTGACTTTAGAGGTTGGAGATTACATCCTCACTCCAGAAATGTGCGTGGAGCGCAAGAGTATCAGTGATTTAATCGGCTCTTTAAATAACGGCCGCCTCTACAGCCAGTGCATCTCCATGTCCCGCTACTACAAGCGTCCCGTGCTTCTGATTGAGTTTGACCCTAGCAAGCCTTTCTCTCTCACTTCCCGAGGTGCCTTGTTTCAGGAGATCTCCAGCAATGACATTAGTTCCAAACTCACTCTTCTTACACTTCACTTCCCCAGACTACGGATTCTCTGGTGCCCCTCTCCTCATGCAACGGCGGAGTTGTTTGAGGAGCTGAAACAAAGCAAGCCACAGCCTGATGCGGCGACAGCACTGGCCATTACAGCAGATTCTGAAACCCTTCCCGAGTCAGAGAAGTATAATCCTGGTCCCCAAGACTTCTTGTTAAAAATGCCAGGGGTGAATGCCAAAAACTGCCGCTCCTTGATGCACCACGTTAAGAACATCGCAGAATTAGCAGCCCTGTCACAAGACGAGCTCACGAGTATTCTGGGGAATGCTGCAAATGCCAAACAGCTTTATGATTTCATTCACACCTCTTTTGCAGAAGTCGTATCAAAAGGAAAAGGGAAAAAGTGA

The mutants of binding sites are highlighted. *WT* wide-type, *mut* mutant

### Cell viability assessment

Cell proliferation was assessed using Cell Counting Kit-8 (CCK-8) assays. Cells were seeded into 96-well plates at a density of 5 × 10^3 cells/plate, with a concentration gradient set for cisplatin intervention (0, 0.2, 0.5, 1, 2, 5, 10, and 20 µg/mL). Following a 48-h incubation period, cells were treated with CCK-8 reagent for 1 h in the dark. The number of viable cells was determined by measuring absorbance at 450 nm using a microplate reader. For the colony formation assay, cells were inoculated onto 6- and 24-well plates with different treatments. In 6-well plates, cells were seeded at a density of 500 cells per well, while 100 cells per well in 24-well plates. After 14 days, colonies were fixed with 4% paraformaldehyde fixation solution (Beyotime, China, cat. P0099) and stained with 0.1% crystal violet staining solution (Beyotime, cat. C0121).

### Comet assay for DNA repair assessment

Following centrifugation and cell counting, cells after different treatment were pipetted into a single-cell suspension at a density of 1 × 10^4 cells/ml. A reagent kit for the single-cell gel electrophoresis assay (Beyotime, China, cat. C2041) was utilized, with all steps executed according to the manufacturer's protocol. After electrophoresis, the gel was stained with propidium iodide (Beyotime, cat. ST1569) and observed using fluorescent microscopy. CASP software was employed for result analysis, with the DNA percentage in the tail serving as descriptors of DNA damage.

### Lactate production and glucose consumption measurements

Lactate and glucose concentrations in the culture supernatants were quantified using a lactate assay kit (Solarbio, China, cat. BC2235) and a glucose assay kit (Solarbio, cat. BC2505), respectively, following the manufacturer’s instructions. Absorbance values were measured at the appropriate wavelengths. The obtained results were normalized by the number of cells in each sample in the culture plates, and lactate production and glucose consumption were calculated by comparison with 10% FBS DMEM/F12.

### Patient derived organoids (PDOs)

Tissue dissociation and organoid culture were performed as previously described [18]. In brief, bladder cancer tissues were washed and minced into small pieces. These tissue fragments were incubated in a digestive medium (containing hepatocyte media with 10 ng/ml EGF, 5% CS-FBS, 10 μM Y-27632, 1 × Glutamax, 100 μg/ml Primocin and collagenase/hyaluronidase) at 37 °C for 10 min. Following digestion, the dissociated tissues were centrifuged and resuspended by TrypLE Express (Invitrogen, USA). The trypsinization process was halted and passed through a 100 μm cell strainer (Corning, USA). The dissociated pellets were centrifuged, resuspended in 60% Matrigel (Corning)/organoid culture media, and plated in a 24-well plate. Once the drops solidified, 1.5 ml of organoid culture media was added to the well, and the medium was refreshed every 3–4 days.

### Immunoblots

Total protein was extracted using radioimmunoprecipitation assay lysis buffer (Beyotime, China). A 10% or 12% sodium dodecyl sulfate‐polyacrylamide gel was chosen for total protein separation, and the proteins were then transferred to nitrocellulose membranes (Millipore, USA). The membranes were cut horizontally and incubated with primary antibodies, including anti-CXCL14 (Affinity, USA, cat. DF12377), anti-fibroblast activation protein (FAP) (Abcam, Britain, cat. ab28244), anti-α-SMA (Abcam, cat. ab7817 and Proteintech, USA, cat. 80,008–1-RR), anti-Bax (Proteintech, cat. 50,599–2-Ig), anti-γH2AX (Proteintech, cat. 29,380–1-AP), anti-STAT3 (Proteintech, cat. 10,253–2-AP), anti-phosphorylated-STAT3 (Tyr705) (Cell Signaling, cat.9145), anti-phospho-JAK2-Y1007/1008 (Abclone, cat. AP0531), anti-JAK2 (Abclone, cat. A7694), anti-ubiquitination (Proteintech, cat. 10,201–2-AP), anti-CCR7 (NOVUS, USA, cat. NBP3-12,012 and Proteintech, cat. 25,898–1-AP), anti-β-actin (Sangon Biotech, China, cat. D110001), anti-excision repair cross-complementation group 4 (ERCC4) (Affinity, cat. AF0295), anti-caspase 3/p17/p19 (Proteintech, cat. 19,677–1-AP), anti-multidrug-resistance protein 2 (MRP2) (Proteintech, cat. 29,261–1-AP), anti-Vimentin (Abcam, cat. ab92547), anti-Fibroblast specific protein 1 (FSP1) (Proteintech, cat. 16,105–1-AP), anti-Hexokinase 2 (HK2) (Proteintech, cat. 22,029–1-AP) and anti-lactate dehydrogenase A (LDHA) (Selleck, USA, cat. F0233).

### Co-immunoprecipitation (Co-IP) and ubiquitination assay

Cells were harvested and lysed using IP lysis buffer (Beyotime, cat. P0013). IP was conducted by incubating Anti-HA beads (MCE, USA, cat. HY-K0201) with the lysate or by overnight incubation of an appropriate antibody with the cell lysate at 4 °C, followed by 4–6 h of incubation with Protein A/G immunoprecipitation beads. Bead-bound proteins were isolated using a magnetic grate. Immunomagnetic beads were then washed three times with cold lysis buffer and eluted with SDS loading buffer by boiling for at least 10 min. IgG (Cell Signaling Technology, cat. 3900S) was employed as the negative control. To detect and inhibit ubiquitin protein, 20 mM anti-ubiquitin (Cell Signaling Technology, cat. 3933) and MG132 dissolved in DMSO were also selected and applied.

### Luciferase reporter assay

The promoter regions of CXCL14 (Table 2) were cloned into a pGL3-basic vector (Promega, USA) with firefly luciferase reporter and transfected into T24 and 293 T cells, along with their respective controls and the PRL-TK plasmid (Promega). Similarly, pcDNA3.1 plasmid containing cDNA encoding either STAT3 or vector was co-transfected into cells. Firefly luciferase activities were detected by the Dual-Luciferase Reporter Assay Kit (Beyotime, cat. RG029S), following the manufacturer’s instructions, with Renilla luciferase serving as the transfection control.

### Extracellular Acidification Rate (ECAR)

ECAR was measured using the Seahorse Bioscience XF24 extracellular flux analyzer (XFe24, USA) to evaluate the glycolytic capacity of the cells, following the standard protocol. Briefly, cells with different pretreatments were seeded onto XF 24-well plates at a density of 2 × 10^4 cells per well and incubated for 16 h. The cells were then treated with 200 µl of Seahorse basic culture medium (Agilent Technologies, Inc.) and placed in a CO_2_-free incubator at 37℃ for 60 min. After calibration with the hydration plate, the XF 24-well plate containing the cells was supplemented with 25 mM glucose, 1 µM oligomycin, and 50 mM 2-DG (all from Seahorse), followed by analysis using the Seahorse energy metabolism analyzer (Agilent Technologies, Inc.).

### Glucose and lactate uptake assay

To evaluate glucose uptake by T24 cells and lactate uptake by fibroblasts, cells were co-cultured in 24-well plates at a density of 1 × 10^4 cells per well for 24 h. Following three washes with PBS, the cells were incubated with 2-NBDG (100 µM; MCE, USA; cat. no. HY-116215) and 2-hydroxypropanoic acid-CY3 (20 µM; XI’AN QIYUE BIOLOGY, China; cat. no. Q-0087622) for 30 min. After an additional three PBS washes, the cells were incubated in complete medium, and the uptake of the two substances was assessed using fluorescence microscopy.

### Real‐time quantitative polymerase chain reaction

Total RNA was extracted from various cells processed by different experiments, and reverse transcription of RNA to cDNA was carried out using a PrimeScript RT reagent kit (TaKaRa, Japan). Primers utilized in the study were designed and synthesized by Tsingke (Beijing, China), and their sequences are provided in Table 1. Real-time quantitative PCR (RT-qPCR) was conducted with SYBR Green (TaKaRa) on an ABI 7500 Real-Time RT-qPCR System (Applied Biosystems), following the manufacturer’s instructions. The mRNA expression levels of different genes were assessed using the quantification approach (2^-ΔΔCt method) relative to the expression levels of β-actin.

### Bioinformatic analysis

Gene expression data from 411 bladder cancer tissue samples were retrieved from The Cancer Genome Atlas (TCGA) database (http://portal.gdc.cancer.gov/). Additionally, data from 149 bladder cancer patients who received adjuvant chemotherapy were retrieved from the Gene Expression Omnibus (GEO) database (GSE169455) (https://www.ncbi.nlm.nih.gov/geo/). Gene expression and clinical information from three additional datasets, comprising 165 patients (GSE13507), 93 patients (GSE31684), and 424 patients (GSE32894), were also obtained from the GEO database for validation. In addition, single-cell sequencing data (GSE145137) from a patient with chemotherapy-resistant MIBC were obtained from the GEO database, with data from murine models excluded from the analysis [19]. The"Seurat"R package was employed for controlling data quality, reducing data dimensionality, and performing cell annotation according to the Seurat manual [20]. Furthermore, the"Monocle"R package was utilized to detect the differentiation trajectories of cells. Gene set enrichment analysis (GSEA) was used to analyze different functional phenotypes between bladder cancer patients with high- and low-expression of CXCL14. Gene set permutations were performed 1000 times to obtain a normalized enrichment score (NES) in each analysis. NES > 1.6 and nominal *p*-value < 0.05 were set as the cutoff criteria. Kyoto encyclopedia of genes and genomes (KEGG) gene sets (c2.cp.kegg.v7.2.symbols.gmt) was obtained from the Molecular Signatures Database. Functional enrichment analysis of gene ontology (GO) and KEGG of the mRNA associated with the fibroblast cluster were completed using R software (https://www.r-project.org/).

### Statistical analysis

All experiments were independently repeated at least three times. The data were analyzed using GraphPad Prism 8.0 software and presented as mean ± standard deviation (SD). Unpaired Student’s *t* tests were performed to compare the differences between two groups. One-way ANOVA followed by Tukey’s multiple comparisons test was utilized for multigroup comparisons. Pearson’s correlation coefficient was employed to determine linear correlations. Categorical variables were compared through chi-squared test. Statistical significance was set at *p* < 0.05.

## Results

### CAFs contribute to chemoresistance in bladder cancer

**Fig. 1:**
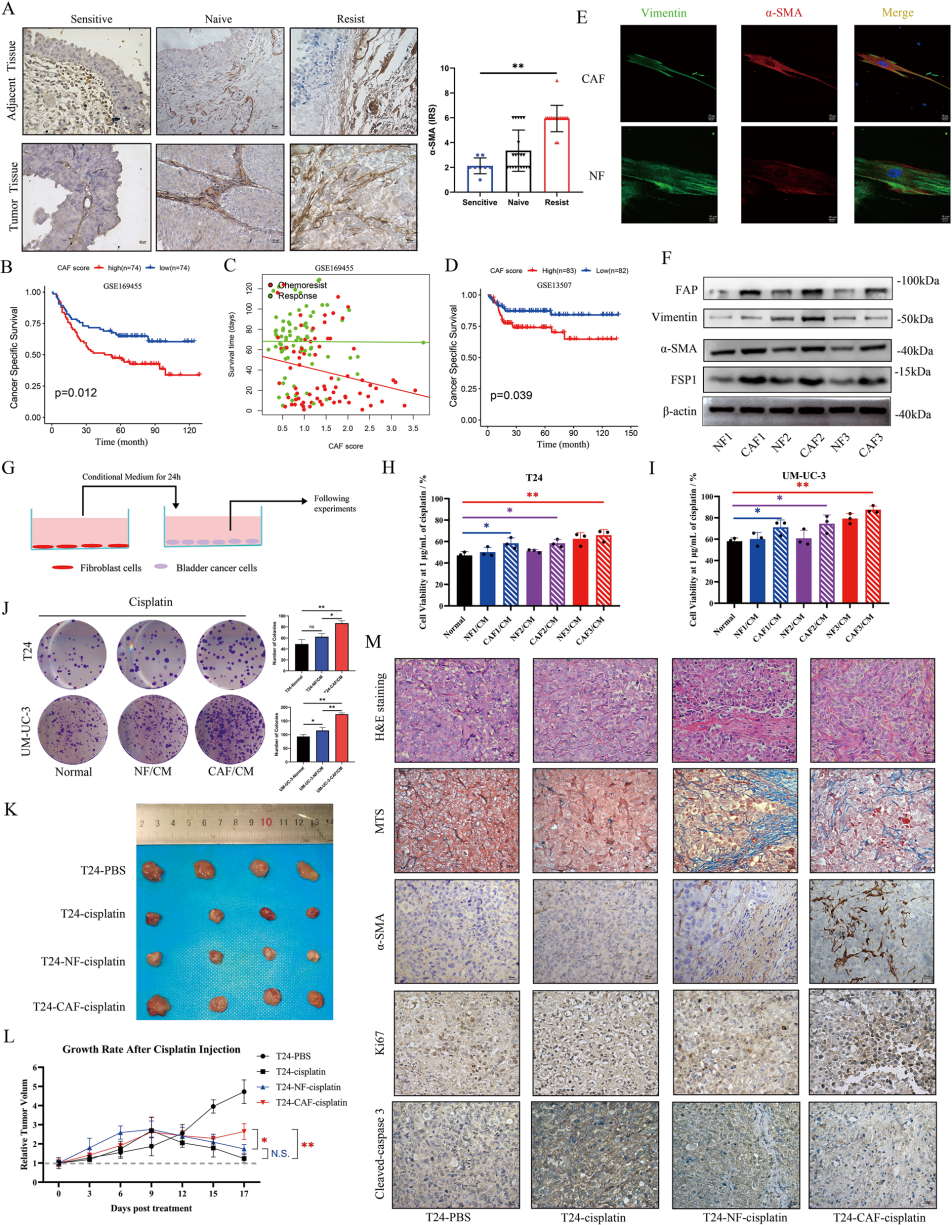
CAFs promote bladder cancer cell resistance to cisplatin in a paracrine manner. **A** Representative IHC images showing α-SMA expression in bladder cancer and adjacent tissues from the three groups. IRS scores were calculated on the basis of α-SMA expression in cancer tissues derived from patients with bladder cancer sensitive to cisplatin (*n* = 8), resistant to cisplatin (*n* = 16) and not receiving treatment before surgery (naïve group, *n* = 20). Scale bar, 20 μm. **B** Kaplan–Meier analysis was conducted to evaluate cancer-specific survival in bladder cancer patients undergoing chemotherapy. **C** Patients were categorized into drug response and chemoresistance groups on the basis of pathological progression. The survival times of the patients and the CAF scores of the samples were then visualized for comparison. **D** Kaplan–Meier analysis was performed to evaluate cancer-specific survival in two groups of bladder cancer patients stratified by CAF score. **E** IF staining of α-SMA and vimentin in CAFs and NFs. Scale bar, 10 μm. **F** The expression of α-SMA, FAP, vimentin and FSP1 in different strains of CAFs and NFs was detected by Western blotting. **G** Schematic depicting the collection of fibroblasts derived CM and maintenance of bladder cancer cells. The cells were incubated in CM for 24 h. **H**, **I** After 48 h of treatment with 1 μg/mL cisplatin, T24 or UM-UC-3 cell viability was measured using a CCK8 assay. **J** Colony numbers of bladder cancer cells cultured in different CMs with 1 μg/mL cisplatin. Colonies containing more than 50 cells were counted. **K** Excised tumor images at the end of the experiment from each group (*n* = 4 per group). **L** The relative tumor growth rate of T24 cells alone or coinjected with NFs (T24-NFs) or CAFs (T24-CAFs) was assessed following the intraperitoneal administration of cisplatin or PBS twice a week. **M** Representative images of HE, Masson and IHC staining of subcutaneous xenograft tissues from different groups. Scale bar, 20 μm**.** The data are presented as the means ± SDs, and the experiments were performed at least three times. The Pearson correlation coefficient was used for correlation analysis. *P* values were determined by unpaired Student’s *t* test or one-way ANOVA followed by Tukey’s test; *P < 0.05 and **P < 0.01

To investigate the TME composition in bladder cancer and adjacent tissues, we conducted IHC to visualize fibroblasts in the interstitial region. IHC staining of α-SMA in 44 patient-derived tissues (represented by one patient as shown in Fig. S1 A, B) revealed a greater proportion of CAFs in tissues from patients resistant to cisplatin treatment (represented by one patient from each group as shown in Fig. 1A). To further validate our findings, we analysed prognostic data from the GSE169455 dataset, which includes 148 bladder cancer patients who received platinum-based chemotherapy. Patients were categorized into high- and low-CAF score groups on the basis of the expression of ACTA2 and CCL19, which are established biomarkers for CAFs, using Cox regression analysis [21, 22]. The results indicated that patients with higher CAF scores had significantly poorer cancer-specific survival and recurrence-free survival rates than did those with lower CAF scores (Fig. 1B and Fig. S1C). Additionally, when patients were divided into response and chemoresistance groups on the basis of pathological progression, the chemoresistance group presented significantly higher CAF scores and shorter survival times than the response group did (Fig. 1C). Regression analysis revealed a negative correlation between the CAF score and survival time in the chemoresistance group (*R* = −10.480, *P* = 0.0496), but no such correlation was observed in the Response group (*R* = −0.2931, *P* = 0.961). To validate our findings, we analysed data from 165 bladder cancer patients in the GSE13507 dataset. Patients in the low CAF score group presented better cancer-specific survival (CSS) and overall survival (OS) rates (Fig. 1D and Fig. S1D). However, no significant difference in recurrence-free survival (RFS) was observed between the two groups (Fig. S1E). Additional validation was performed using data from two independent cohorts: 93 patients in the GSE31684 dataset and 424 patients in the GSE32894 dataset. Consistent with our findings, cancer-specific survival (CSS) analysis further confirmed that bladder cancer patients with higher CAF scores were associated with poorer prognoses (Fig. S1F, G).

**Scheme 1:**
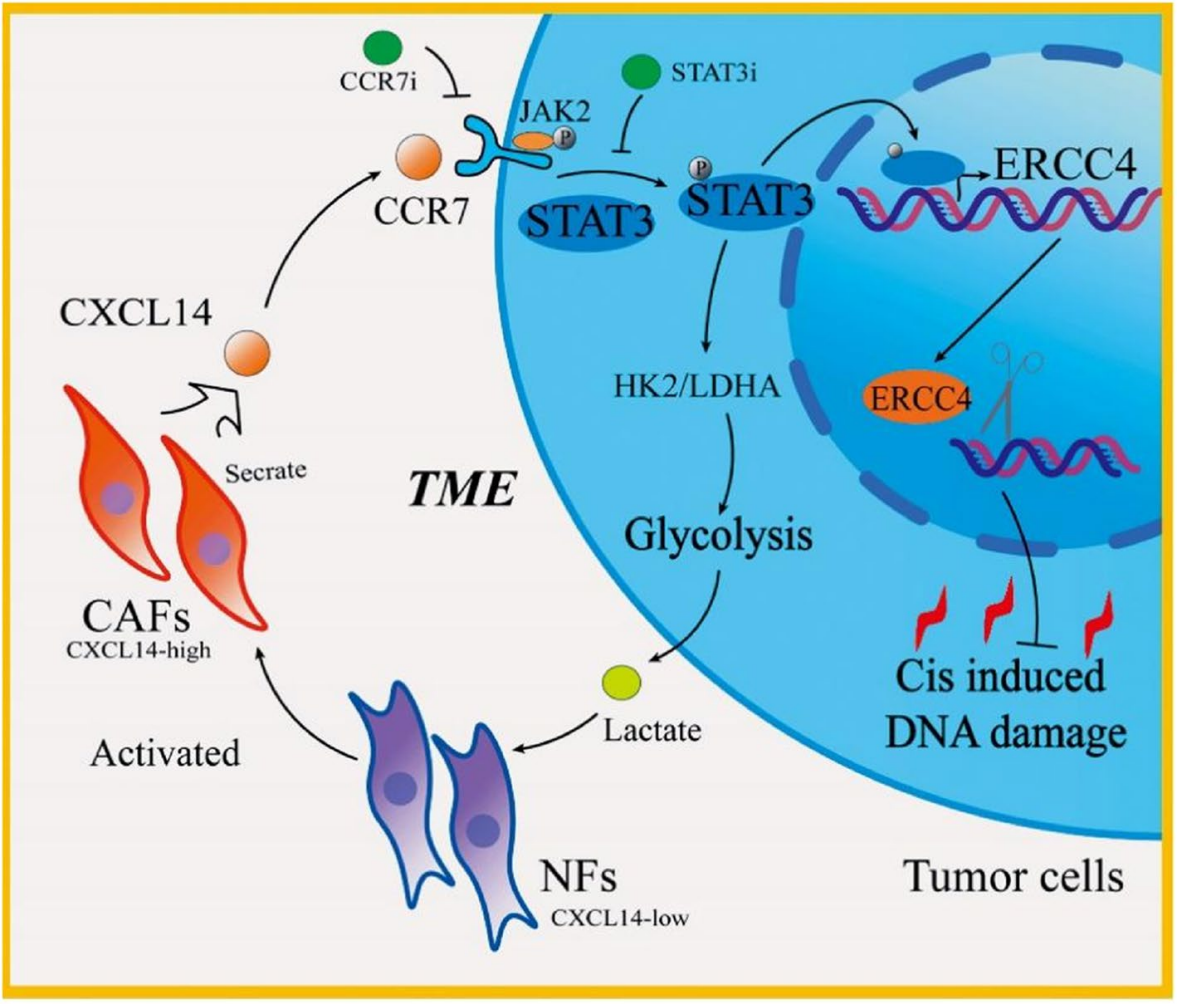
Schematic diagram

Primary fibroblasts were subsequently isolated from both bladder cancer tissues and adjacent normal bladder tissues, and the purity of the CAF were further confirmed by flow cytometry (Fig. S1 H). Immunofluorescence and Western blot analysis confirmed the characteristic markers of these fibroblasts. Both normal fibroblasts (NFs) and CAFs were positive for vimentin (Fig. 1E), an interstitial cell marker, but CAFs presented increased expression of activated fibroblast markers, including FAP, FSP1, and α-SMA (Fig. 1F). Recent evidence has suggested that CAFs can promote chemoresistance in cancer cells through paracrine signaling, although reports in bladder cancer are limited [12, 23]. In this study, we collected conditioned medium (CM) from both CAFs and NFs, and tested the sensitivity of T24 and UM-UC-3 cells to cisplatin after different treatments (as shown in the schematic diagram (Scheme 1), Fig. 1G). Compared with those in the NF/CM or medium control groups, the half maximal inhibitory concentration (IC50) in the CAF/CM-treated group was nearly two to four times greater (Fig. 1H, I and Fig. S1I, J). To assess the long-term effects of CM treatment, we performed a colony formation assay under 1 μg/mL cisplatin, which revealed that CAF/CM significantly enhanced the chemoresistance of bladder cancer cells in spheroids (Fig. 1J). The TME is a complex system characterized by intricate cellular compositions and interactions. To validate our findings in vivo, we investigated the impact of CAFs on cisplatin resistance in bladder cancer xenografts. Following the coinjection of bladder cancer cells with either NFs or CAFs subcutaneously, the mice were treated with four weeks of cisplatin or PBS via intraperitoneal injection. The animals were subsequently euthanized, and the xenografts were dissected. Our results revealed larger tumor size (Fig. 1K) and accelerated tumor growth (Fig. 1L and Fig. S1K) in mice coinjected with CAFs than in those in the T24- only or NF-coinjection groups. Histological analyses, including H&E, Masson’s trichrome, and α-SMA immunohistochemical staining, revealed increased extracellular matrix deposition in xenografts coinjected with CAFs (Fig. 1M). In addition, Ki67 IHC staining demonstrated enhanced tumor cell proliferation in the presence of CAFs under cisplatin treatment, whereas cleaved caspase-3 staining indicated a marked reduction in cisplatin-induced apoptosis in the CAF coinjection group (Fig. 1M). Conversely, NFs did not exhibit any protective effect on bladder cancer cells under cisplatin treatment.

Our findings indicate the widespread distribution of CAFs in bladder cancer tissues especially in patients with chemoresistance. Upon isolation, CAFs playe an assisting role in the development of chemoresistance in bladder cancer cells in a paracrine manner.

### CAF/CM-induced upregulation of ERCC4 reduces the ability of cisplatin to trigger DNA damage

**Fig. 2:**
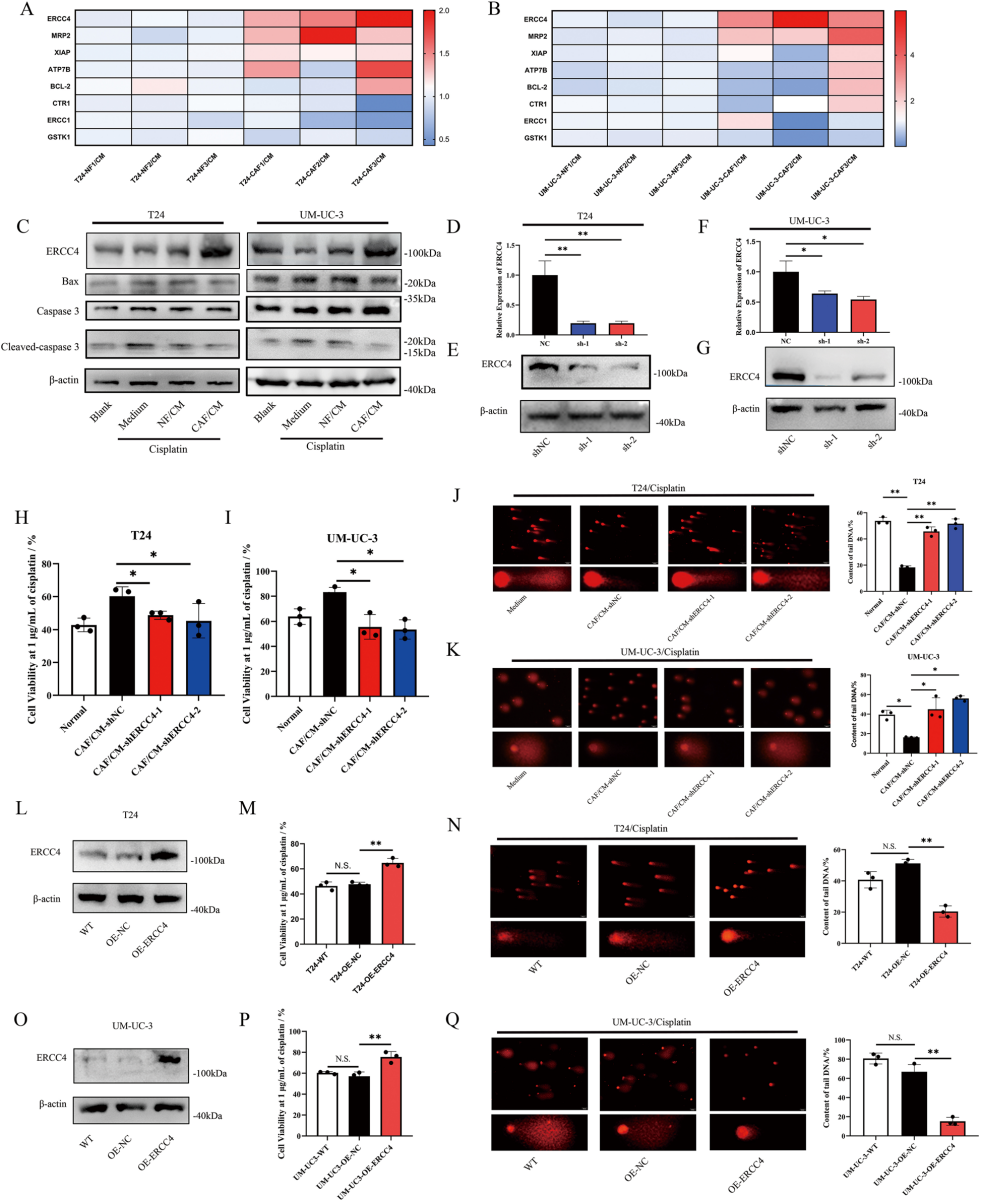
CAFs/CM regulate the development of chemoresistance in bladder cancer cells by upregulating ERCC4 expression. **A**, **B** The relative expression of key regulators in T24 and UM-UC-3 cells incubated with NF/CM or CAF/CM from different patients was detected by RT‒qPCR, and all the expression levels were relative to those in the paired NF/CM group. **C** The expression of ERCC4, Bax and cleaved-caspase 3 (relative to caspase 3) in T24 and UM-UC-3 cells subjected to different CM treatments was detected by Western blotting. **D**, **F** RT-qPCR analysis of the transcription levels of ERCC4 after transfection with different shRNAs. ACTB was chosen as the internal reference. **E**, **G** Western blot analysis was used to assess the translational expression and intervention efficiency of ERCC4 in T24 cells transfected with different shRNAs. **H**, **I** After 48 h of treatment with 1 μg/mL cisplatin, T24 or UM-UC-3 cell viability after transfection with different shRNAs was measured by a CCK8 assay. **J**, **K** Representative images of DNA fragments in T24 and UM-UC-3 cells after different treatments. Tail DNA contents were calculated by CASP software and served as descriptors of DNA damage. Scale bar, 100 μm**. L**, **O** Western blot analysis was used to assess the overexpression efficiency of ERCC4 in T24 cells transfected with OE-NC or OE-ERCC4. **M**, **P** After 48 h of treatment with different concentrations of cisplatin, T24 or UM-UC-3 cell viability after transfection with different lentiviruses was measured by a CCK8 assay. **N**, **Q** Representative images of DNA fragments from T24 and UM-UC-3 cells transfected with different lentiviruses after cisplatin treatment. The total DNA content was calculated and is presented as the mean ± SD. Scale bar, 10 μm**.** *P* values were determined by unpaired Student’s *t* test or one-way ANOVA followed by Tukey’s test; *P < 0.05 and **P < 0.01

The mechanisms contributing to cisplatin resistance in cancer cells include increased drug efflux through proteins such as MRP2 and ATP7B, enhanced detoxification via GSTK1, inhibited drug uptake through CTR1, altered DNA repair involving ERCC1 and ERCC4, and increased production of antiapoptotic proteins such as Bcl-2 and XIAP [24, 25]. Consequently, we examined the expression of these key regulators in bladder cancer cells to investigate the mechanism triggered by CAFs. As shown in Figure 2A and B, both ERCC4 and MRP2 were upregulated at the transcriptional level following CAF/CM treatment in T24 and UM-UC-3 cells. Western blot analysis further confirmed the overexpression of ERCC4 and MRP2 in these two cell lines after CAF/CM treatment (Fig. 2C and Fig. S2 A, B). Additionally, after treatment with 1 μg/mL cisplatin, we observed that compared with the medium control or NF/CM, the CAF/CM significantly inhibited drug-induced apoptosis (Fig. 2C). To explore the roles of ERCC4 and MRP2 in the development of drug resistance in bladder cancer cells, we modulated their expression in T24 and UM-UC-3 cells (Fig. 2D, F and Fig. S2 E, G) and confirmed the effects of their knockdown at the translational level (Fig. 2E, G and Fig. S2 F, H). We subsequently assessed the sensitivity of these cells to cisplatin following incubation with CAF/CM. Our results demonstrated that the suppression of ERCC4 expression mitigated the chemoresistance induced by CAF/CM (Fig. 2H, I and Fig. S2 C, D), whereas the inhibition of MRP2 had no such effect (Fig. S2 I, J). ERCC4, also known as XPF, is a component of the ERCC1-XPF structure-specific endonuclease and plays a crucial role in DDR processes including nucleotide excision repair (NER), interstrand crosslink (ICL) repair, and homologous recombination (HR) repair [26]. ERCC4 overexpression in cells has been reported to reduce the degree of DNA damage and subsequent apoptosis induced by ICL-containing compound [27]. We assessed DNA damage in bladder cancer cells following different treatments, and the results revealed that CAF/CM significantly inhibited cisplatin-induced DNA damage (Fig. 2J, K and Fig. S2K, L). To further investigate the role of ERCC4 in chemoresistance, we overexpressed ERCC4 in both cell lines (Fig. 2L, O). The overexpression of ERCC4 led to decreased sensitivity to cisplatin (Fig. 2M, P and Fig. S2M, N) and reduced DNA damage (Fig. 2N, Q and Fig. S2O, P) in bladder cancer cells. These findings highlight the potential role of secretory factors from CAFs in inducing chemoresistance in bladder cancer cells, primarily through the transcriptional regulation of ERCC4 overexpression.

### CXCL14 sourced from CAFs is a contributor to the chemoresistance of bladder cancer

**Fig. 3:**
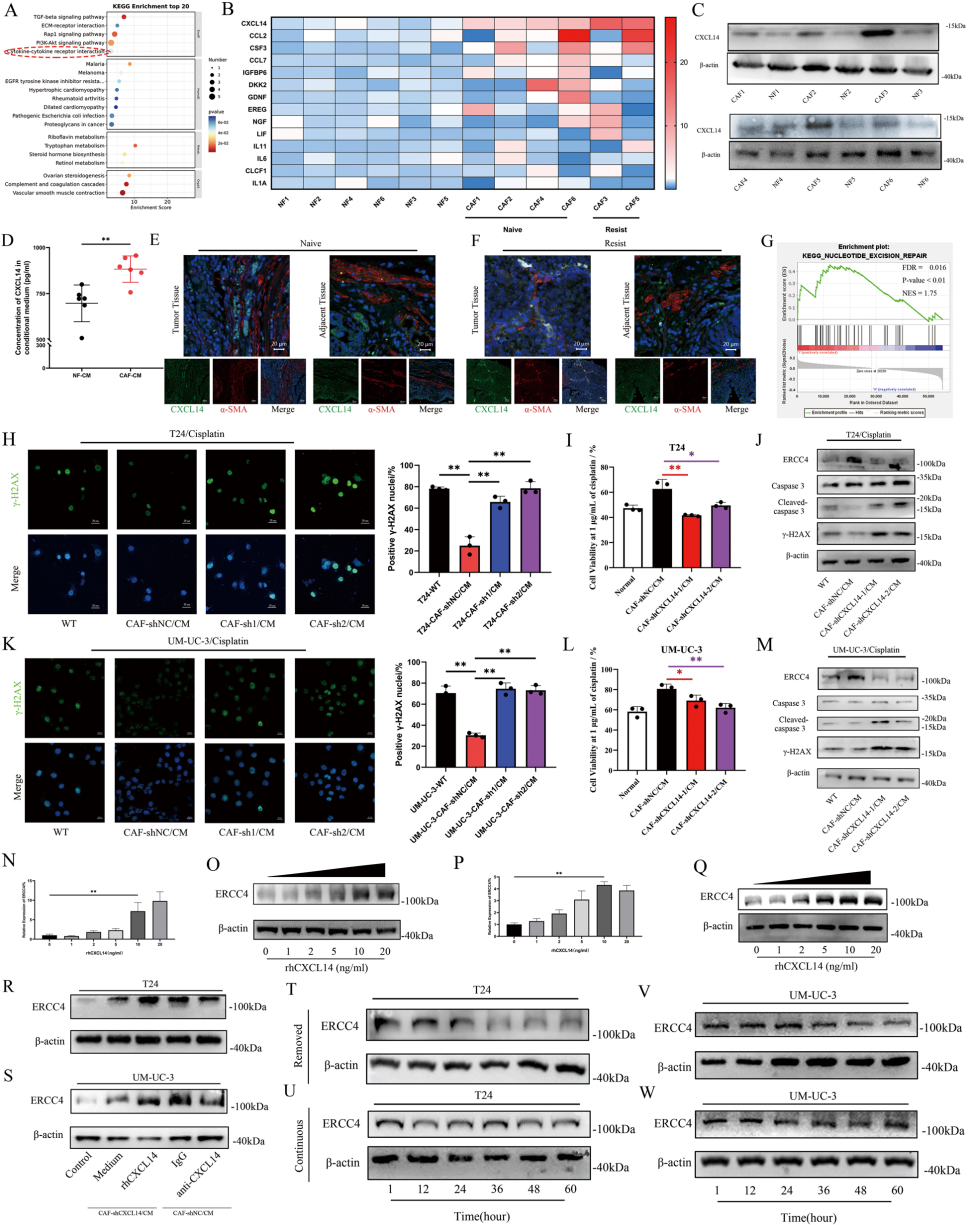
Exogenous CXCL14 contributes to chemoresistance in bladder cancer in a dose dependent manner. **A** KEGG analysis revealed the enriched terms associated with the feature genes of CAFs. **B** RT-PCR was used to determine the expression levels of secretory cytokines in CAFs from different patients, and all the expression levels were relative to those in the paired NF group. **C** Expression of CXCL14 was examined by Western blot in six paired primary fibroblast samples. **D** ELISA measurement of CXCL14 in the supernatants of 6 CAFs and NFs. **E**, **F** IF staining of bladder cancer tissues and paired adjacent tissues for α-SMA and CXCL14. Scale bar, 100 μm for 100 × magnification and 20 μm for the 200 × magnification**. G** GSEA plots illustrating KEGG terms enriched in the CXCL14 high-expression group, which are particularly important in nucleotide excision repair. **H**, **K** Representative IF image showing the expression of γH2AX in T24 and UM-UC-3 cells, with percentages of positive nuclei (termed 10 foci or more per nucleus) counted per sample on the right. Scale bar, 20 μm. **I**, **L** After 48 h of treatment with various concentrations of cisplatin, the viability of the different groups cultured in different conditioned media was assessed using a CCK8 assay. **J**, **M** ERCC4, γH2AX, and cleaved caspase-3 protein levels were examined by Western blot in T24 and UM-UC-3 cells cultured in different conditioned media supplemented with 1 μg/mL cisplatin. **N**, **P** ERCC4 mRNA expression in T24 and UM-UC-3 cells was quantified by RT-qPCR following treatment with various concentrations of rhCXCL14. **O**, **Q** Changes in ERCC4 protein expression in response to rhCXCL14 stimulation were evaluated in T24 and UM-UC-3 cells. **R**, **S** ERCC4 expression was assessed in T24 and UM-UC-3 cells indirectly cocultured with various CAF subtypes and treated with either rhCXCL14 or an anti-CXCL14 neutralizing antibody. **T**, **U** The effect of continuous rhCXCL14 exposure versus withdrawal on ERCC4 expression was determined in T24 cells. **V**, **W** Similarly, ERCC4 expression was measured in UM-UC-3 cells under sustained rhCXCL14 treatment or after its removal. The data are presented as the mean ± SD, and the experiments were performed at least three times. *P* values were determined by unpaired Student’s *t* test or one-way ANOVA followed by Tukey’s test; *P < 0.05 and **P < 0.01

To investigate the crucial factor(s) that trigger chemoresistance and ERCC4 overexpression, we analysed single-cell RNA sequencing data from a patient with chemotherapy-resistant muscle-invasive urothelial bladder cancer [19]. Nine clusters were identified and classified into different subtypes according to marker genes (Fig. S3 A). We then extracted fibroblast subtypes and used KEGG/GO enrichment analysis to investigate the potential functional characteristics of chemoresistant CAFs. The results indicated that CAFs may function in a “Cytokine-cytokine receptor interaction” manner (Fig. 3A). To identify crucial factors, we analysed the expression profiles of secretory and inflammatory cytokines in CAFs [28, 29] and detected their expression in NFs and CAFs isolated from six paired bladder cancer and adjacent normal tissues. We found that CXCL14 was generally overexpressed in these six sets of paired fibroblasts, especially in patient 3 and patient 5 sourced CAFs, which were patients diagnosed with clinical chemoresistance after neoadjuvant chemotherapy (Fig. 3B). To further understand the relationship between CAFs and CXCL14, we extracted the forementioned chemoresistance-related fibroblast single-cell RNA sequencing data and regrouped them.

By analyzing the differentially expressed genes between different cell populations, we found that these CAFs could be divided into three clusters (Fig. S3 B). GO functional analysis was used to explore the functional characteristics of those subtypes. The biological process of Cluster 0 was enriched mainly in “T cell mediated immunity”, that of Cluster 1 was enriched mainly in “extracellular matrix organization”, and that of Cluster 2 was enriched mainly in “animal organ morphogenesis” (Fig. S3 C). Pseudotime analysis revealed that Cluster 2 was projected onto the root, Cluster 0 was projected onto the trunk, and Cluster 1 was projected onto two branches, one as a single branch and another differentiated from part of Cluster 0 (Fig. S3 D, E). As mentioned above, previous studies have reported that CAFs can be categorized into various subsets on the basis of their specific functions and several biomarkers, including inflammatory CAFs (iCAFs) and myofibroblastic CAFs (myCAFs) [30]. While the precise functions of each subgroup remain unclear, we identified distinct gene signatures for different subsets during our analysis. Notably, the gene signatures of Cluster 0 coincided with certain markers associated with iCAFs, such as SDC1, CD70 and LIF. The gene signatures of Cluster 1 aligned with specific markers of myCAFs, whereas the corresponding CAF subgroup was not identified in Cluster 2. By integrating our findings from pseudotime and GO enrichment analyses, we identified this particular subgroup as being involved in the initiation and activation of CAFs and therefore referred to it as naïve CAFs (Fig. S3 F). These results indicate the potential of naïve CAFs as the initial strain that grows into iCAFs and then differentiates into iCAFs and myCAFs. Therefore, we detected the expression of CXCL14 in those three clusters, and found that CXCL14 was overexpressed in both the iCAF and naïve-CAF subtypes (Fig. S3 G, H). In combination with previous findings that CXCL14 overexpression is consistent with chemoresistance in bladder cancer patients, we classified abnormal CXCL14 expressing CAFs as differentiated iCAFs or undifferentiated naïve CAFs.

To further investigate the role and function of CXCL14 in bladder cancer progression, we evaluated the translational expression of CXCL14 in the aforementioned patient-derived tissues. Western blot analysis revealed significant upregulation of CXCL14 in CAFs, particularly in patients exhibiting chemoresistance (Fig. 3C). By measuring the concentration of CXCL14 in the CM of six paired fibroblasts, we observed that compared with NFs, CAFs produced and secreted more CXCL14 (Fig. 3D). To further explore the distribution of CXCL14 expression within the TME, immunofluorescence staining was conducted for α-SMA and CXCL14 in tissue samples from Patient 1 and Patient 3. The results revealed that CAFs were the primary source of CXCL14 in tumor tissues, with significantly elevated expression levels observed in chemoresistant patients (Fig. 3E, F). To explore the role of CXCL14 in the development of chemoresistance in bladder cancer, we performed GSEA using RNA-seq data from 414 bladder cancer patients in the TCGA database. The analysis revealed that CXCL14 is functionally enriched not only in the"bladder cancer"pathway but also in several cell cycle and DNA damage repair-related pathways, including"base excision repair,""mismatch repair,""nucleotide excision repair,"and"homologous recombination"(Fig. 3G and Fig. S3 I). Additionally, Pearson regression analysis of TCGA data revealed a significant correlation between CXCL14 expression and pathways associated with the"cell cycle"and"DNA damage"(Fig. S3 J, K). These findings suggest that CXCL14 may play a role in modulating DNA damage repair, which is consistent with the known functions of ERCC4.

Therefore, we next examined whether deficient expression of CXCL14 in CAFs affects cisplatin induced DNA damage in bladder cancer cells. We inhibited CXCL14 expression in CAFs using lentivirus transfection. The efficiency of the lentivirus was validated at the transcriptional and translational levels (Fig S3L, M), and the secretion of CXCL14 in CAFs was significantly inhibited (Fig.S3N). We then treated bladder cancer cells with CM from different groups, and immunofluorescence staining of γH2AX revealed decreased DNA damage in T24 and UM-UC-3 cells incubated with CAFs/CM, which lost their protective role after CXCL14 expression was modulated (Fig. 3H, K). Cell viability assays indicated that CAF/CM-induced chemoresistance to cisplatin in bladder cancer cells was significantly blocked after CXCL14 was intervened in CAFs (Fig. 3I, L and Fig. S3 O, P). Western blot analysis revealed that deficient expression of CXCL14 in CAFs failed to increase the expression of ERCC4 and inhibited DNA damage-induced apoptosis in bladder cancer cells (Fig. 3J, M). These findings indicated that CAF-derived CXCL14 promotes the development of chemoresistance in bladder cancer cells by enhancing ERCC4-mediated DNA damage repair. We used recombinant human CXCL14 (rhCXCL14) to simulate the effect of exogenous CXCL14 on bladder cancer cells. The results revealed that rhCXCL14 treatment significantly increased both the transcriptional and translational expression of ERCC4 in T24 and UM-UC-3 cells in a concentration-dependent manner (Fig. 3N-Q). Therefore, we identified 10 ng/ml as the optimal concentration. To assess the duration of CXCL14 action in the tumor microenvironment and its potential for reversing drug resistance, we blocked CXCL14 in CAF/CM by adding either IgG or a CXCL14 neutralizing antibody. Western blot analysis revealed that exogenous CXCL14 significantly increased ERCC4 expression in bladder cancer cells in CXCL14-deficient CAFs. However, this effect was notably inhibited by the addition of the CXCL14 neutralizing antibody in the NC group (Fig. 3R, S). Additionally, by comparing ERCC4 expression in different bladder cancer cell lines after continuous treatment and subsequent rhCXCL14 removal, we observed that rhCXCL14 consistently promoted ERCC4 overexpression, but this effect was maintained for only 24 h after rhCXCL14 was removed (Fig. 3T-W).

In summary, our results demonstrate that CXCL14 derived from CAFs within tumor tissues enhances cellular DNA damage repair in a paracrine manner, and its effect on ERCC4 is dose-dependent.

### CXCL14 enhances ERCC4 transcription by the CCR7/STAT3 axis

**Fig. 4:**
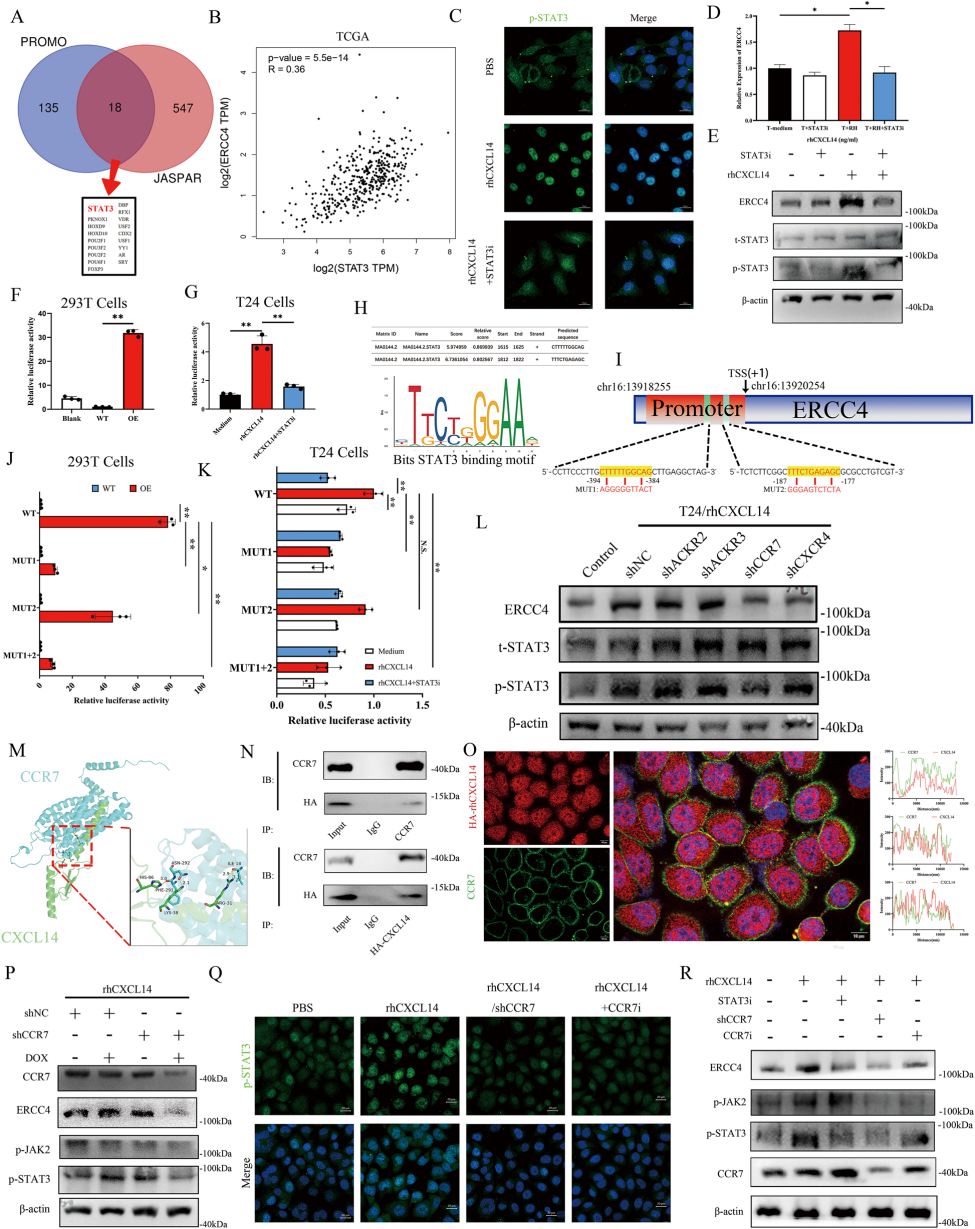
The CXCL14/CCR7 signaling axis enhances ERCC4 transcription through JAK2/STAT3 activation and transcriptional regulation. **A** Venn diagram showing the overlapping promotors of ERCC4 predicted by JASPAR (https://jaspar.genereg.net/) and PROMO (https://alggen.lsi.upc.es/). **B** Correlation analysis between STAT3 and ERCC4 (GEPIA, http://gepia.cancer-pku.cn/). **C** Representative immunofluorescence images showing the expression and distribution of p-STAT3 in T24 cells following treatment with exogenous CXCL14 or STAT3i. Scale bar, 20 μm. **D** RT-qPCR analysis was used to detect ERCC4 expression in T24 cells subjected to different treatments, with ACTB as the internal reference. **E** Western blot analysis of ERCC4, STAT3, and phosphorylated STAT3 expression in T24 cells treated with the rhCXCL14 protein or a STAT3 inhibitor. **F** The luciferase activities of the ERCC4 promoter containing sequences in STAT3 overexpressing 293 T cells were assessed. **G** Luciferase activity in T24 cells was assessed after treatment with rhCXCL14 or STAT3i. **H** STAT3 binding elements at the promoter region of ERCC4 were predicted by JASPAR. **I** A schematic representation of the ERCC4 promoter sequence and its mutated variants, which were individually or jointly cloned and inserted into the pGL3 vector for the luciferase reporter assay. **J**, **K** The luciferase activities of the ERCC4 promoter containing sequences in different groups of T24 and 293 T cells were assessed. **L** Western blot analysis of ERCC4 expression and STAT3 phosphorylation in T24 cells inhibited by different receptors. **M** Molecular docking simulation studies were conducted to explore the interactions between CCR7 and ERCC4. **N** The interactions between CXCL14 and CCR7 were confirmed with co-IP, followed by Western blot analysis. **O** Immunofluorescence images of HA-tagged CXCL14(red), CCR7 (green), and DAPI (blue) in T24 cells. The intensity profiles of CXCL14 (red lines) and CCR7 (green lines) colocalization signals are shown as plotted lines at three random sites. Scale bar, 10 μm. **P** The inducible knockdown efficiency of CCR7, along with the expression levels of ERCC4, phosphorylated JAK2, and phosphorylated STAT3, was evaluated in T24 cells subjected to different treatment conditions. **Q** Representative immunofluorescence images showing the expression and subcellular localization of phosphorylated STAT3 in T24 cells subjected to various treatments. Scale bar, 20 μm. **R** The expression of ERCC4, CCR7, p-JAK2, and p-STAT3 was analysed in T24 cells following treatment with specific inhibitors or CCR7-targeting shRNAs. The data are presented as the means ± SDs, and the experiments were performed at least three times. *P* values were determined by unpaired Student’s *t* test or one-way ANOVA followed by Tukey’s test (*P < 0.05 and **P < 0.01), and the Pearson correlation coefficient was used for correlation analysis

After the promoters of ERCC4 were scanned by JASPAR (https://jaspar.genereg.net/) and PROMO (https://alggen.lsi.upc.es/), we found that 18 promoters overlapped (Fig. 4A). Given that signaling pathways play important roles in the transcriptional modulation of key cellular regulators, we identified STAT3 as a potential transcriptional regulator of ERCC4, whichwas positively correlated with ERCC4 expression in bladder cancer patients in the TCGA cohort (Fig. 4B). With the use of the ENCODE database (www.encodeproject.org), we detected binding peaks of STAT3 in the promoter regions of ERCC4 in three types of cancer cell lines (Fig.S4A). Immunofluorescence results confirmed that rhCXCL14 activated p-STAT3 and facilitated its nuclear translocation, whereas inhibition of STAT3 activity in T24 cells using STAT3i impaired this activation (Fig. 4C). Following the blockade of CXCL14-induced STAT3 activation, we observed subsequent transcriptional inhibition of ERCC4 expression (Fig. 4D, E). To investigate whether STAT3 binds to the promoter region of ERCC4 and how CXCL14 enhances its expression in bladder cancer cells, we overexpressed STAT3 in 293 T cells (Fig. S4B) as an external validation and modulated STAT3 phosphorylation in T24 cells with rhCXCL14 or rhCXCL14 combined with STAT3i (Fig. S4C). We cloned the ERCC4 promoter into the pGL3 vector and transfected this construct into various cell lines. The results revealed a significant increase in luciferase activity in STAT3-overexpressing 293 T cells (Fig. 4F). Additionally, we observed that STAT3 activation by rhCXCL14 significantly increased the luciferase activity of the ERCC4 promoter, while STAT3i effectively blocked this process (Fig. 4G). These findings further confirmed that CXCL14 elevates ERCC4 transcription through STAT3. We analysed four STAT3 chromatin immunoprecipitation sequencing (ChIP-seq) datasets across three different cancer types and identified two prominent STAT3 binding peaks within the ERCC4 promoter region (Fig. S4D). Further motif analysis using the JASPAR database predicted two potential STAT3 binding sites on the ERCC4 promoter (Fig. 4H). To confirm these binding sites, we introduced mutations at these sites individually or together in the ERCC4 promoter vector (Fig. 4I) and transfected these plasmids into different cell lines. Compared with that in the wild-type group, luciferase activity was significantly lower in the cells transfected with mutations at site 1 and at both sites 1 and 2 (Fig. 4J, K). Mutation at site 2 also decreased the fluorescence intensity but to a lesser extent than mutation at site 1 did, indicating that STAT3 primarily enhances ERCC4 expression by binding to the promoter region at the −384 to −394 site upstream of the transcriptional start site.

To study the molecular mechanisms underlying STAT3 activation, we analysed intermediate ligands or receptors of STAT3 that may be activated by CXCL14, such as the cell membrane receptor CCR7 (Fig. S4E). Compared with previously reported CXCL14 receptors including ACKR2, ACKR3, and CXCR4 [15, 30], CCR7 knockdown resulted in the most pronounced inhibition of CXCL14-induced STAT3 activation and ERCC4 protein expression (Fig. 4L, and Fig. S4 F-I). Molecular docking simulations were conducted to examine the interactions between CCR7 and CXCL14. Among the ten docking models with docking scores below 270, the top models were identified, with a docking score of 270 between CCR7 and CXCL14 and a confidence score of 96% (Fig. 4M). We found that the amino acid residue HIS86 of the CXCL14 protein can bind to the amino acid residue PHE291 of the CCR7 protein via a hydrogen bond of 3.0 Å, whereas LYS38 of CXCL14 can bind to ASN292 of the CCR7 protein through a hydrogen bond of 2.1 Å. Additionally, ARG31 of the CXCL14 protein forms two hydrogen bonds, measuring 3.1 Å and 2.9 Å, with the ILE18 residue of the CCR7 protein (Fig. S4J). We confirmed the interaction between CCR7 and CXCL14 in T24 cells through coimmunoprecipitation assays (Fig. 4N). Additionally, the interaction between CXCL14 and CCR7 in bladder cancer cells was further validated by fluorescence confocal microscopy (Fig. 4O).

We further confirmed the role of CCR7 in mediating CXCL14 induced ERCC4 overexpression. Following CCR7 knockdown, the activation of STAT3 and the rhCXCL14-induced upregulation of ERCC4 transcription in T24 cells were significantly inhibited (Fig. S4K-M). Cytokines typically bind to receptor subunits, initiating receptor activation and subsequent phosphorylation of Janus kinases. Activated Jak2 then phosphorylates tyrosine residues on the receptor, creating a docking site for STAT3, which is phosphorylated at Tyr705, leading to the activation of STAT3 homodimers. To further investigate the role of CCR7 in mediating CXCL14-induced ERCC4 overexpression and elucidate the associated signaling pathways, we employed the Tet-on system to induce CCR7 knockdown in T24 cells. Upon doxycycline induction, the rhCXCL14-induced activation of JAK2/STAT3 phosphorylation and ERCC4 overexpression were significantly inhibited (Fig. 4P and Fig. S5A). As a small molecule inhibitor, Cmp2105 was first reported to inhibit CCR7 activation by competitively binding to its ligand [32]. After CCR7i treatment or CCR7 knockdown, CXCL14 induced STAT3 activation was effectively blocked (Fig. 4Q). We further confirmed that CXCL14 enhances ERCC4 expression in T24 cells through binding to CCR7 and activating STAT3 phosphorylation (Fig. 4R and Fig. S5 B).

Collectively, our results suggest that CAF-derived CXCL14 targets CCR7 in bladder cancer cells, enhancing ERCC4 transcription through JAK2/STAT3 activation and subsequently binding to the ERCC4 promoter region.

### Disruption of the CXCL14/CCR7/JAK2/STAT3/ERCC4 axis increases the sensitivity of bladder cancer cells to cisplatin in vitro and in vivo

**Fig. 5:**
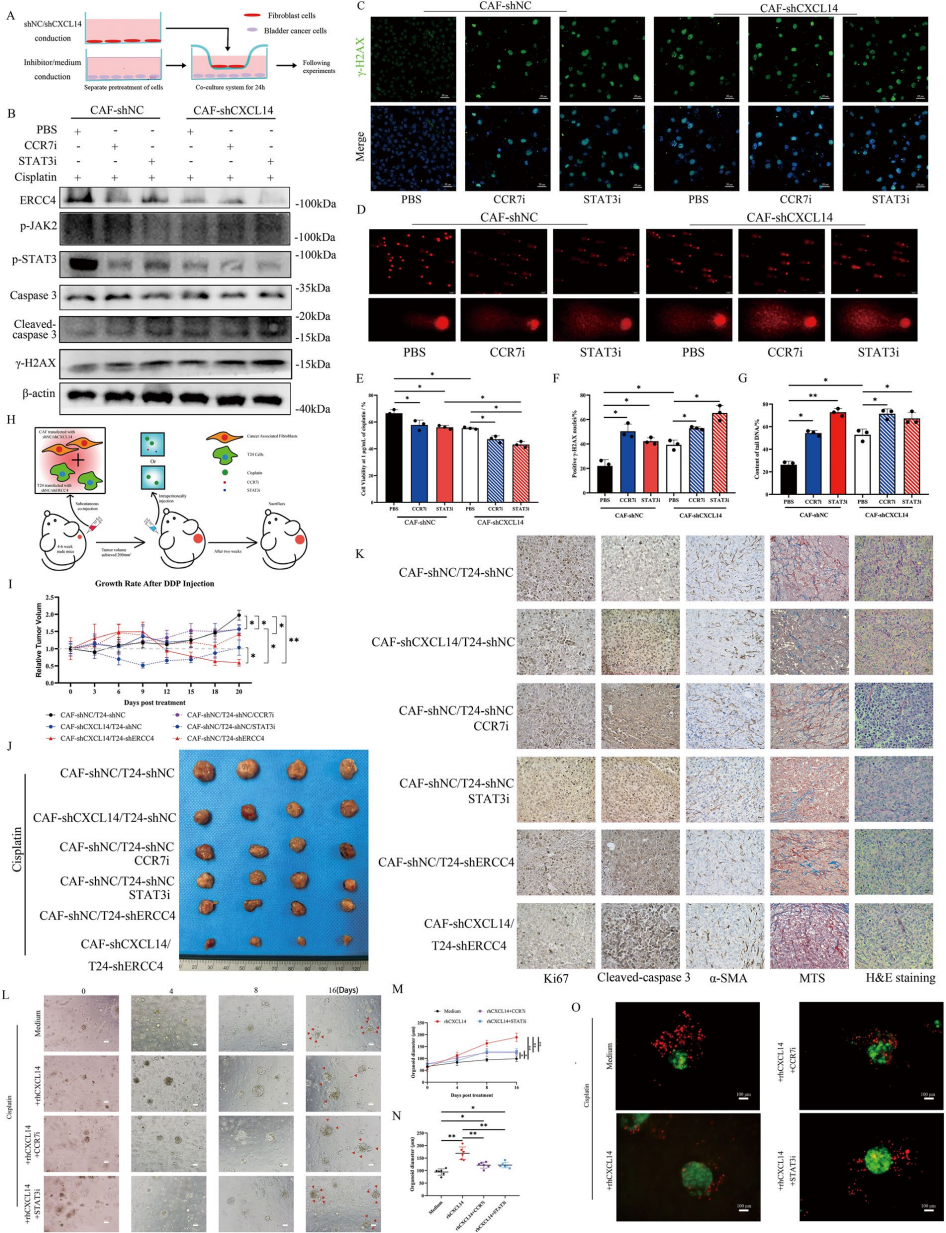
Disruption of the CXCL14/CCR7/JAK2/STAT3 axis increases the sensitivity of bladder cancer cells to cisplatin. **A** Schematic illustration of the untouched coculture system of CAFs and T24 cells. The cells were pretreated with different transfections and utilized for further experiments after coculture for 24 h. **B** Protein levels of ERCC4, CCR7, γH2AX, cleaved caspase-3, STAT3, and phosphorylated STAT3 were evaluated by Western blot in T24 cells treated with various media or inhibitors in the presence of 1 μg/mL cisplatin. **C** Representative immunofluorescence images showing γH2AX expression in T24 cells under different treatment conditions. Scale bar, 20 μm. **D** Representative images illustrating DNA fragmentation in T24 cells following different treatments. Scale bar, 100 μm. **E** Cell viability in each treatment group was assessed using the CCK-8 assay after 48 h of cisplatin exposure. **F** Quantification of γH2AX-positive nuclei (defined as ≥ 10 foci per nucleus) on the basis of the results shown in Fig. 5C. **G** Tail DNA content, derived from Fig. 5D, was used as an indicator of DNA damage severity. **H** Schematic depicting the coinjection of CAFs and T24 cells with different shRNAs and the intraperitoneal injection of cisplatin in the presence or absence of the CCR7 or STAT3 inhibitor. **I** The relative tumor growth rate of T24 cells transfected with different shRNAs and coinjected with CAFs deficient in CXCL14 expression or negative controls. Tumor-bearing mice were intraperitoneally administered cisplatin along with medium or inhibitors twice a week, starting when the average tumor volume reached approximately 200 mm^3 (indicated by arrows). **J** Representative gross tumor images from each treatment group. **K** Representative images of HE, Masson and IHC staining of subcutaneous xenograft tissues from different groups. Scale bar, 20 μm. **L** Representative bright-field images of bladder cancer patient-derived organoids subjected to different treatments in the presence of 1 μg/mL cisplatin (the red arrows indicate apoptotic cells). Scale bar, 100 μm. **M** Line graphs depicting the growth of tumor organoids in each treatment group. **N** Organoid size monitored after treatment for 12 days. **O** AO/PI staining allowed for the simultaneous detection of live and dead cells in the organoid lines. Green fluorescence represented live cells, whereas red fluorescence represents dead cells. Scale bar, 100 μm. The data are presented as the means ± SDs, and the experiments were performed at least three times. *P* values were determined by unpaired Student’s *t* test or one-way ANOVA followed by Tukey’s test; *P < 0.05 and **P < 0.01

To further confirm whether CAFs regulate bladder cancer cell chemoresistance through CCR7 induced STAT3 activation, we employed CAFs infected with shNC or shCXCL14. The cancer cells were then divided into different groups with a contactless coculture system as shown in Fig. 5A. By detecting the expression of ERCC4 and the activity of STAT3, we confirmed the ability of our inhibitors to suppress STAT3 phosphorylation and ERCC4 transcription (Fig. 5B and Fig.S5 C). We detected decreased expression of ERCC4 after CXCL14/CCR7/JAK2/STAT3 axis blockade and increased cleaved-caspase 3 and γ-H2AX expression (Fig. 5B). The proportion of γH2AX-positive T24 cells under different treatment conditions indicated that DNA damage levels were significantly elevated when CXCL14 was absent or when CCR7/STAT3 was inhibited (Fig. 5C, F). Comet assays conducted under alkaline conditions with 1 μg/mL cisplatin demonstrated that disrupting any component of the CXCL14/CCR7/STAT3 signaling axis between CAFs and T24 cells effectively hindered the DNA damage repair process (Fig. 5D, G). We found that cancer cells cocultured with CAFs deficient in CXCL14 expression presented increased sensitivity to cisplatin. Additionally, the application of CCR7 or STAT3 inhibitors further reduced the development of chemoresistance, particularly at a cisplatin concentration of 1 μg/mL (Fig. 5E and Fig.S5D). To further confirm the paracrine role of CXCL14 from CAFs in chemoresistance induction in bladder cancer cells, and to explore whether other signaling mechanisms, such as extracellular vesicles or direct contact, could exert similar effects, we cocultured T24 cells with shNC/shCXCL14 CAFs for 24 h (Fig.S5E). We subsequently sorted the CAFs via flow cytometry using PE-FAP and maintained the T24 cells to assess their sensitivity to cisplatin. However, no significant changes were observed in T24 cells cocultured with shCXCL14 CAFs (Fig. S5F). To investigate the in vivo effects of CXCL14 knockdown or blockade of the CXCL14/CCR7/STAT3 axis within the tumor microenvironment (TME), we coinjected CAFs and T24 cells, each with different shRNAs, subcutaneously into nude mice. This was followed by intraperitoneal injections of cisplatin combined with either CCR7i, STAT3i, or PBS (Fig. 5H). Our findings demonstrated that CXCL14 knockdown in CAFs significantly enhanced tumor sensitivity to cisplatin, an effect that was also observed in the CCR7i and STAT3i groups (Fig. 5I and Fig. S5G). Moreover, the combination of CXCL14 knockdown in CAFs with ERCC4 knockdown in T24 cells resulted in the most substantial tumor growth inhibition. Additionally, ERCC4 knockdown in T24 cells alone increased the tumor sensitivity to cisplatin and reduced tumor burden in the bladder cancer model (Fig. 5J). Histological analysis with H&E staining, Masson’s trichrome staining, and α-SMA immunohistochemical staining illustrated the stromal architecture of the xenograft tumors. Ki67 and cleaved-caspase 3 immunohistochemical staining indicated increased apoptosis and decreased proliferation of cancer cells following cisplatin treatment upon blockade of the CXCL14/CCR7/STAT3/ERCC4 axis (Fig. 5K).

To investigate the impact of exogenous CXCL14 on the chemoresistance of bladder cancer, we collected and cultured patient-derived organoids (PDOs) from a bladder cancer patient who underwent radical cystectomy without prior therapy to assess the clinical relevance of our findings. These PDOs exhibited solid structures in vitro, and our results demonstrated that treatment with rhCXCL14 enhanced PDO growth even in the presence of cisplatin (Fig. 5L). However, the addition of STAT3 or CCR7 inhibitors in combination with cisplatin effectively arrested organoid growth (Fig. 5M, N), with an increased proportion of apoptotic cells (Fig. 5O), highlighting the potential of these inhibitors to counteract the development of chemoresistance.

Taken together, our results suggest that disrupting the CXCL14/CCR7/STAT3 signaling axis, both in vitro and in vivo, enhances DNA damage and increases cisplatin sensitivity in bladder cancer.

### Resistant bladder cancer cells convert fibroblasts to CAFs by lactate production

**Fig. 6:**
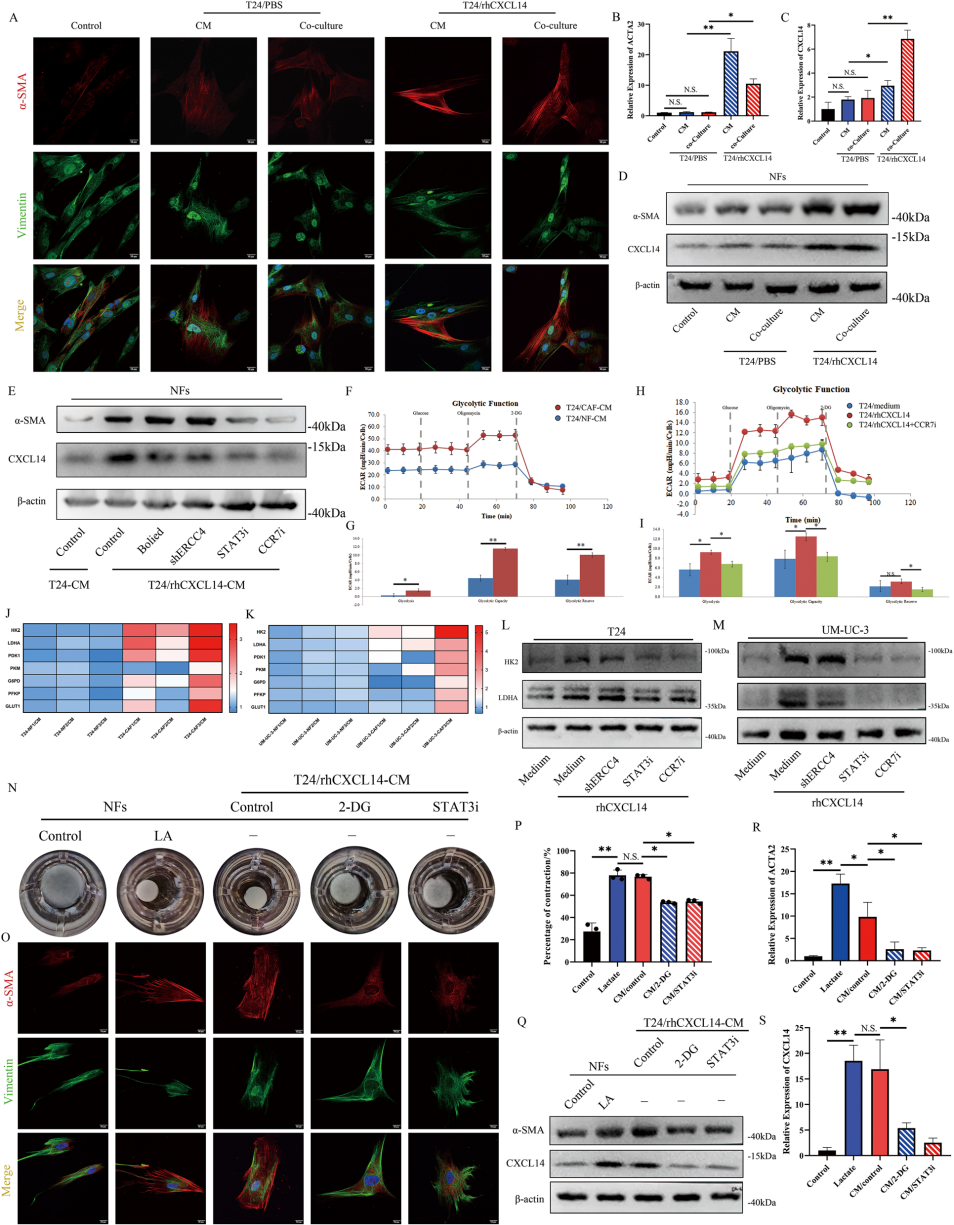
CXCL14-induced chemoresistant cells convert fibroblasts into CAFs through lactate secretion. **A** IF staining of α-SMA and vimentin in NFs subjected to different treatments for 24 h. Scale bar, 10 μm. **B**, **C** RT-qPCR analysis was performed to evaluate the transcription levels of ACTA2 and CXCL14 in NFs following coculture with T24 cells or exposure to CM with different pretreatments, and the levels were further detected at the translational level. **D** Western blotting was used to assess the expression of α-SMA and CXCL14 in normal fibroblasts subjected to various conditioned medium treatments. **E** Western blot results demonstrated normal fibroblast activation under different inhibitor treatments. **F** The ECAR of T24 cells pretreated with NF/CM or CAF/CM was detected by Seahorse analysis. **G** Glycolysis level, capacity and reversal ability of T24 cells subjected to different treatments were measured by the Seahorse assay. **H** The ECAR of T24 cells pretreated with rhCXCL14 or CCR7i was detected by Seahorse analysis. **I** Glycolysis level, capacity and reversal ability of T24 cells subjected to different treatments were measured by the Seahorse assay. **J**, **K** The mRNA expression of key glycolysis genes were examined by RT-qPCR in T24 and UM-UC-3 cells subjected to different CAF-/NF-CM treatments. **L**, **M** Western blot analysis was performed to assess HK2 and LDHA expression in T24 and UM-UC-3 cells following exposure to various inhibitors or shRNAs targeting the CCR7/STAT3/ERCC4 axis. **N**, **P** A collagen contraction assay was used to assess the contraction ability of NFs treated as indicated, and the results were quantified using ImageJ software. **O** Representative immunofluorescence images showing α-SMA and vimentin expression in NFs treated as indicated. Scale bar, 10 μm. **Q** Western blot analysis of the expression of α-SMA and CXCL14 in NFs subjected to various treatments was performed to assess their activation. Additionally, RT-qPCR was conducted to verify activation at the transcriptional level (**R**, **S**). The data are presented as the means ± SDs, and the experiments were performed at least three times. *P* values based on unpaired Student’s *t* test or one-way ANOVA followed by Tukey’s test; *P < 0.05 and **P < 0.01

After clarifying the role of CAF-derived chemokines in the regulation of DNA damage repair in cancer cells, we next investigated whether there is a regulatory effect of chemoresistant cells on normal fibroblasts. Interestingly, both the coculture system and the conditioned medium from rhCXCL14 pretreated T24 cells significantly increased the expression of α-SMA (Fig. 6A). RT-qPCR and western blot results demonstrated the activation of NFs to CAFs from resistant T24 cells in a paracrine manner (Fig. 6B-D).

Given the extensive involvement of metabolic and signal transduction pathways in tumor cells, we aimed to identify the key components of T24 cells responsible for CAF activation and to determine whether the CXCL14/CCR7/STAT3/ERCC4 signaling axis is involved in this process. To this end, we denatured the protein components in the supernatant at high temperatures and treated T24 cells with various inhibitors or shRNAs. The supernatant was then collected and applied to normal fibroblasts. Western blot analysis revealed that STAT3i and CCR7i effectively blocked the activation induced by the chemoresistant T24 supernatant (Fig. 6E). Additionally, the activated substance was found to be thermostable, suggesting that it is a metabolite rather than a protein signaling molecule. The Warburg effect, a defining characteristic of malignant tumor cell metabolism, is critically involved in tumor drug resistance. To further investigate whether the acidic microenvironment or glucose metabolites are responsible for the activation of NFs into CAFs, the pH of the culture medium was adjusted to 6.8 using hydrochloric acid to simulate an acidic microenvironment [33]. Additionally, lactic acid and pyruvate were added as representative glucose metabolites for comparison. Western blot analysis and fibroblast contractility measurements demonstrated that lactate was the only metabolite capable of effectively inducing fibroblast activation and enhancing CXCL14 expression (Fig. S6A, B). Recent studies have shown that lactate produced by tumor cells can affect homeostasis, contribute to immunosuppression, and promote fibroblast activation within the TME [34], but research on how chemoresistant cancer cells influence the activation of CAFs in bladder cancer is limited. To address this, we first examined the glucose consumption and lactate production rates of T24 cells under different fibroblast cocultures. The results indicated significantly elevated glycolytic levels in cells treated with CAF/CM (Fig. S6 C). Additionally, ECAR analysis revealed greater glycolytic activity and capacity in T24 cells indirectly cocultured with CAFs than in those cocultured with NFs (Fig. 6F, G). Fluorescently labelled glucose and lactate were utilized to further investigate changes in glucose metabolism and lactate utilization between different cell types. As shown in Supplementary Fig. 6D, T24 cells cocultured with NFs presented lower glucose uptake than those cocultured with CAFs did, while lactate absorption was significantly greater in CAFs than in NFs. Upon rhCXCL14 treatment, glucose uptake by T24 cells cocultured with NFs was significantly increased (Fig. S6 E), and NFs presented a slight increase in lactate absorption, although not as pronounced as in CAFs, possibly due to the limited treatment duration (Fig. S6 F). Conversely, CAFs co-cultured with T24 cells demonstrated reduced glucose uptake upon CCR7i application. These findings suggest that CXCL14 derived from CAFs enhances glucose uptake in T24 cells and that lactate reabsorption occurs not only in cancer cells but also in activated CAFs.

To investigate the role of the CXCL14/CCR7/STAT3/ERCC4 axis in promoting glycolysis, we evaluated the effects of rhCXCL14, shERCC4, and various inhibitors on glycolytic activity. The results showed that rhCXCL14 enhanced glucose consumption and lactate production in T24 cells. Inhibition of STAT3 and CCR7, but not ERCC4, significantly mitigated these glycolytic changes and capacities (Fig. 6 6H, I and Fig. S6 G). To further elucidate the underlying regulatory mechanisms, we analysed key glycolytic enzymes in T24 and UM-UC-3 cells after 24 h of treatment with different CAF/CM. The results revealed that the aberrant overexpression of HK2 and LDHA was responsible for the increase in glycolysis observed (Fig. 6J, K). Consistently, rhCXCL14 treatment further increased HK2 and LDHA expression. This effect was suppressed by inhibiting STAT3 and CCR7, but not ERCC4 (Fig. 6L, M).

Next, we examined whether enhanced glycolysis and lactate efflux in tumor cells are key drivers of fibroblast activation. When NFs were treated with exogenous lactate or conditioned medium from rhCXCL14-pretreated T24 cells, we observed a significant increase in fibroblast contractility (Fig. 6N, P) and α-SMA expression (Fig. 6O). In contrast, fibroblast activation was markedly inhibited when glycolysis or STAT3 inhibitors were applied. These observations were further supported by RT-qPCR and Western blot analyses, which confirmed changes at both the transcriptional (Fig. 6R, S) and translational levels (Fig. 6Q).

We cocultured NFs with CAFs directly or indirectly to determine whether CAFs can activate NFs independently of cancer cells. Western blot and immunofluorescence analyses demonstrated that NFs did not undergo activation or CXCL14 expression induction, regardless of the coculture method (Fig. S6H, I).

These results elucidate a mechanism whereby CAFs promote the activation of NFs by inducing aberrant glycolysis and lactate excretion in bladder cancer cells. Lactate produced by chemoresistant cancer cells not only enhances fibroblast activation and CXCL14 expression but also establishes a positive feedback loop that drives the development of chemoresistance within the TME.

## Discussion

Cisplatin is a cornerstone first-line therapy for patients ineligible for surgery and is widely used to treat various cancers, including bladder cancer. However, resistance to cisplatin remains a major challenge and a significant cause of treatment failure, necessitating alternatives [35]. Emerging evidence underscores the pivotal role of CAFs in shaping the TME and driving tumor chemoresistance through the secretion of various factors. Targeting cytokines that contribute to resistance against platinum-based chemotherapy within the TME is a promising strategy to increase drug response rates in patients with bladder cancer [5]. In our study, we isolated primary fibroblasts from clinical specimens and classified them as NFs or CAFs the basis of their origin and characteristics. We specifically focused on the overexpression and secretion of CXCL14 by CAFs, revealing its role in mediating NER responses in bladder cancer cells, which ultimately facilitates the development of chemoresistance.

In the process of characterizing the subset of CAFs with CXCL14 overexpression, we noticed that they were mainly clustered in the iCAF and naïve-CAF subsets, which is also consistent with reports of prostate cancer [36]. While we observed this effect in our study, it is important to note that the extracted CAFs were not further sorted, and the subtypes of CAFs and their subsequent characteristics will be further explored in our future experiments. CXCL14 is reported to play dual roles in cancer progression, and both its antitumor role and protumor role depend on the cancer type and source [15]. Stromal CXCL14, but not tumor CXCL14, is reported to promote progression [16], and CAF-secreted CXCL14 has been reported to promote tumor growth and invasion of breast and prostate cancer cells [16, 37]. While little is known about its role in bladder cancer, we observed prevalent expression of CXCL14 in bladder tissues, particularly within normal bladder epithelial cells. Conversely, CXCL14 was highly expressed in the tumor stroma, and CAFs obtained from chemoresistant patients presented elevated CXCL14 expression. Additionally, for the first time, we found that exogenous CXCL14 from CAFs could enhance the NER response of bladder cancer cells by upregulating ERCC4 expression.

Eukaryotic cells possess DNA repair pathways that maintain genome stability; however, these mechanisms could also reduce the efficacy of chemotherapy in cancer cells. Platinum-based chemotherapy primarily induces DNA damage to intrastrand G-G dimers, leading to distortion of the DNA helix, inhibition of replication and transcription, and ultimately, apoptosis [38]. Abnormal DNA damage repair in tumor cells, particularly through the NER pathway, plays a central role in removing helix-distorting lesions, including ICLs [26]. NER involves incisions by structure-specific endonucleases, resulting in excision and removal of a single-strand DNA fragment from the damaged DNA strand [39]. In our study, we observed transcriptional upregulation of ERCC4 in CXCL14-induced chemoresistant bladder cancer cells. ERCC4 is a versatile NER protein that functions by cleaving damaged DNA chains in the 5'region [40]. Through the use of CAF/CM and exogenous recombinant proteins, we confirmed that CXCL14 enhanced ERCC4 transcription by the CCR7/STAT3 axis.

Unlike members of the traditional chemokine family, the receptor targeted by CXCL14 is not well characterized. CXCR4, a commonly reported receptor for CXCL14, has been implicated in the regulation of tumor metastasis in renal cell carcinoma and endometrial cancer [31]. Additionally, ACKR2 has been identified as a receptor for exogenous CXCL14 in lung and breast cancers, where it contributes to the activation of tumor cell invasion and migration [15, 41]. In our study, we observed that CXCL14 expression was significantly elevated in the stroma of bladder cancer tissues compared with adjacent tissues, with a more pronounced increase in cisplatin-resistant tissues. These findings suggest that CXCL14 from CAFs may activate the NER response in bladder cancer cells, although the exact mechanism remains unclear. Notably, knocking down CXCR4, ACKR2, or ACKR3 did not significantly inhibit STAT3 activation or reduce ERCC4 expression. Through bioinformatics predictions combined with experimental validation, we identified a novel CXCL14 receptor, a G protein-coupled receptor named CCR7, which appears to be a critical mediator of CAF-driven chemoresistance in bladder cancer cells. Consistent with this finding, the use of small molecule inhibitors to competitively inhibit CCR7 or its downstream effector STAT3 effectively suppressed CXCL14-mediated ERCC4 transcription.

Given the complexity and bidirectionality of signaling interactions within the TME, we further explored whether CXCL14-induced chemoresistant bladder cancer cells contribute to fibroblast activation. Using a noncontact coculture system or supernatant alone, we observed significant activation of normal fibroblasts, which was accompanied by increased CXCL14 expression. To rule out efficiency components, we used high-temperature denaturation, revealing that metabolites, rather than proteins, were responsible for this activation. Given the important role of glucose metabolic reprogramming in tumor progression, the effects of glucose metabolites and the acidic microenvironment on NF activation were investigated. Our results demonstrated that the CXCL14/CCR7/STAT3 axis significantly promotes the glycolytic shift in bladder cancer cells by upregulating the expression of HK2 and LDHA, two key glycolytic enzymes. Furthermore, lactate generated via this signaling pathway effectively facilitates the transformation of normal fibroblasts into CAFs. Lactate has traditionally been regarded as the end product of glycolysis; however, emerging evidence highlights its role as both a carbon source for cellular metabolism and a signaling molecule within the TME [42]. On the one hand, lactate derived from CAFs can fuel breast cancer cell growth or enhance lipid metabolic reprogramming, thereby facilitating cancer metastasis, which often referred to as the reversed “Warburg” effect [43]. On the other hand, as the primary source of lactate within the TME, cancer cells secrete large amounts of lactate to create an acidic microenvironment that suppresses immune cell infiltration [44]. Fibroblasts reuse this lactate, especially under conditions of nutrient restriction induced by tumor cells, to promote their activation [45]. Lactate derived from lung cancer cells can be taken up by CAFs, leading to their activation and subsequent recruitment of macrophages [34]. With the use of C13-labelled metabolic flux analysis, Bhagat et al. revealed that lactate produced by glycolytic pancreatic cancer cells is absorbed and further metabolized by CAFs, resulting in increased 5-hydroxymethylcytosine levels and decreased 5-methylcytosine levels, ultimately driving CAF activation and differentiation [46]. Our study has several limitations in this regard. We did not further investigate the precise mechanisms underlying lactate-induced CAF activation, but we observed an association between CAF activation and increased lactate absorption. The involvement of lactate transporters, such as those in the MCT family, in regulating lactate uptake and their potential role in modulating fibroblast activation remain to be fully elucidated. Addressing this question will be a key focus of our future research.

In conclusion, we elucidated a mechanism by which CAFs contribute to chemoresistance in bladder cancer cells through CXCL14 paracrine signaling (Scheme 1). Specifically, CXCL14 activates the CCR7/JAK2/STAT3 axis, leading to the upregulation of ERCC4 transcription, which promotes DNA damage repair in bladder cancer cells, thereby facilitating the development of chemoresistance. Additionally, exogenous CXCL14 upregulates HK2/LDHA expression via STAT3 activation, resulting in lactate efflux which further converts normal fibroblasts into CAFs. We also demonstrated that small molecule inhibitors, including the STAT3 inhibitor S3I-201 and the CCR7 inhibitor Cmp2105, effectively block the development of drug resistance by targeting different points within the CXCL14/CCR7/JAK2/STAT3 axis, both in vitro and in vivo. This inhibition shows potential for enhancing the efficacy of tumor chemotherapy and offers a novel therapeutic strategy for clinical application.

## Supplementary Information

Supplementary Material 1. Figure S1 A), B) Representative images illustrated E-cadherin and Vimentin expression in bladder cancer and adjacent tissues. Scale bar, indicates 100 μm for the 100 ×, and 20 μm for the 40 ×. C) Kaplan–Meier analysis was conducted to evaluate recurrence-free survival in bladder cancer patients undergoing chemotherapy. D), E) Kaplan–Meier analysis was conducted to evaluate overall survival and recurrence-free survival in two groups of bladder cancer patients stratified by CAF scores. F), G) Kaplan–Meier analysis was performed to evaluate cancer-specific survival (CSS) in two groups of bladder cancer patients stratified by CAF scores from two independent cohorts: 93 patients in the GSE31684 dataset and 424 patients in the GSE32894 dataset, respectively. H) Representative flow cytometry plots (left) demonstrating fibroblast event populations in primary extracted fibroblast cells. I), J) After 48 h treatment of cisplatin with different concentration, viabilities of T24 or UM-UC-3 cell with different conditional medium treatment and cisplatin were measured by CCK8 assay. K) Tumor growth curve of T24 cells alone or co-injected with NFs (T24-NFs) or CAFs (T24-CAFs). Tumor-bearing mice were intraperitoneally administered with cisplatin or PBS twice a week since the average tumor volume reached approximately 200 mm^3 (indicated by arrows). Categorical variables were compared through chi-squared test. Figure S2 A), B) The expression of MRP2 in T24 and UM-UC-3 cells with different conditional medium treatment were detected by Western blot. C), D) After 48 h treatment of cisplatin with different concentration, T24 or UM-UC-3 cell viabilities after cultured in different CM were measured by CCK8 assay. E), G) RT-qPCR analysis detecting MRP2 expression after different shRNAs transfection in T24 and UM-UC-3 cells. F), H) Western blot analysis assessed the translational expression of MRP2 in T24 and UM-UC-3 cells. I), J) After 48 h cisplatin treatment, viabilities of T24 or UM-UC-3 cells with different CM treatment were measured by CCK8 assay. K), L) Tail length was calculated using CASP software to evaluate the DNA damage levels in different groups of T24 and UM-UC-3 cells. M), N) After 48 h cisplatin treatment, viabilities of T24 or UM-UC-3 cells with different lentivirus transfections were measured by CCK8 assay. O), P) Tail length was calculated using CASP software to evaluate the DNA damage levels in different groups of T24 and UM-UC-3 cells under treatment of cisplatin. *P* values based on unpaired Student’s *t* test or one-way ANOVA followed by Tukey’s test; *P < 0.05 and **P < 0.01. Figure S3 A) TSNE dimensionality reduction of all cells showing CAFs in chemoresistant bladder cancer samples, and re-clustering of CAFs identified three subpopulations. B) Heatmap demonstrated marker genes for each cluster. C) GO enrichment analysis demonstrated the enriched terms by feature genes of different subclusters. D), E) Pseudotime analysis demonstrated that cluster 2 was projected onto the root, cluster 0 was projected onto the trunk while cluster 1 were projected onto two branches, one as single and another differentiated with part of cluster 0. F) Representative images illustrated feature genes expression level in each cluster. G), H) CXCL14 exhibited high expression levels in both cluster 0 and 2. I) GSEA plots illustrating KEGG terms enriched in CXCL14 high-expression group, of particular significance in DDR-related pathways including “bladder cancer”, “base excision repair”, “mismatch repair”, “nucleotide excision repair” and “homologous recombination”. J), K) Pearson regression analysis of the TCGA data to access the correlation between CXCL14 expression and"Cell cycle"and"DNA damage"pathways. L) RT-qPCR analyzed the transcriptional expression of CXCL14 after transfection with different shRNAs in CAFs, with ACTB as the loading control. M) Western blot analysis assessing the translational expression of CXCL14 in CAFs transfected with different shRNAs. N) ELISA measurement of CXCL14 concentration in the CM of CAFs transfected with different shRNAs. O), P) After 48 h treatment of cisplatin with different concentration, T24 or UM-UC-3 cell viabilities after cultured in different CM were measured by CCK8 assay. Data were presented as the means ± SDs, and experiments were performed at least three times. *P* values based on unpaired Student’s *t* test or one-way ANOVA followed by Tukey’s test; *P < 0.05 and **P < 0.01. Categorical variables were compared through chi-squared test, and Pearson correlation coefficient was used for correlation analysis. Figure S4 A) STAT3 binding peaks within the promoter region of ERCC4 were visualized using the ENCODE database (www.encodeproject.org) in GM12878, MCF-10A, and HeLa-S3 cells (highlighted in blue and indicated by red arrows). B) STAT3 overexpression in 293 T cells was confirmed by Western blot. C) Activation of STAT3 by rhCXCL14 and its inhibition by a STAT3 inhibitor (STAT3i) in T24 cells were demonstrated via Western blot analysis. D) IGV exhibiting seven CHIP-seq data sourced from three type of cancer including B cell lymphoma, T cell lymphoma and breast cancer, demonstrated the enrichment of STAT3 in the promoter of ERCC4 (highlighted in blue). E) The protein interaction network was predicted by STRING (https://cn.string-db.org/). F-I) RT-qPCR analysis detecting efficiency of different shRNA’s inhibition on andidate receptors for CXCL14 including ACKR2, ACKR3, CXCR4, and CCR7 in T24 cells. J) The specific binding sites of CCR7 and CXCL14 were predicted using AutoDock Vina(https://vina.scripps.edu/). K) Western blot analysis of ERCC4, CCR7, STAT3, and phosphorylated STAT3 expression in T24 cells treated with recombinant human CXCL14, with or without CCR7 knockdown. L), M) RT-qPCR analysis detected ERCC4 and CCR7 expression in T24 cells under different treatment. Data were presented as the means ± SDs, and experiments were performed at least three times. *P* values based on unpaired Student’s *t* test or one-way ANOVA followed by Tukey’s test; *P < 0.05 and **P < 0.01. Categorical variables were compared through chi-squared test, and Pearson correlation coefficient was used for correlation analysis. Figure S5 A) Western blotting was conducted to analyze the expression of STAT3 and JAK2 in T24 cells with or without inducible knockdown of CCR7. B) Western blot analysis examining total STAT3 and JAK2 expression in T24 cells under different treatment of inhibitors or knockdown of CCR7. C) Western blot analysis examining STAT3 and JAK2 expression in T24 cells of different groups in the presence of 1 μg/mL cisplatin. D) After 48 h of cisplatin treatment, T24 cell viability in different groups was assessed using the CCK-8 assay. E) Representative images of T24 cells co-cultured with CAFs transfected with different lentiviruses. F) Following co-culture, FAP-positive fibroblasts were sorted using flow cytometry, while T24 cells without FAP expression were retained for subsequent analyses. G) After 48 h of cisplatin treatment, the viability of sorted T24 cells co-cultured with different CAFs was assessed using the CCK-8 assay. H) Tumor growth curves of T24 cells with different shRNA transfections coinjected with CAFs deficient in CXCL14 expression or negative control. Tumor-bearing mice were intraperitoneally administered cisplatin along with medium or inhibitors twice a week, starting when the average tumor volume reached approximately 200 mm^3 (indicated by arrows). Data were presented as the means ± SDs, and experiments were performed at least three times. *P* values based on unpaired Student’s *t* test or one-way ANOVA followed by Tukey’s test (*P < 0.05 and **P < 0.01). Figure S6 A) Western blot analysis of CXCL14 and α-SMA expression in NFs exposed to different metabolites or simulated acidic microenvironments B) Collagen contraction assay was assessed for the contraction ability of NFs treated as indicated, and results were quantified using ImageJ software. C) Changes in glucose and lactate levels in the cell supernatant, reflecting the rates of glucose consumption and lactate production in T24 cells treated with different CAFs and paired NFs, with each group compared to its corresponding NF. D) Representative images illustrating glucose uptake (green fluorescence) and lactate uptake (red fluorescence) in T24 cells cocultured with NFs or CAFs and stimulated with various drugs. White light images were included to observe cell morphology and distinguish between cell types. Scale bar, 50 μm. E), F) Immunofluorescence quantification of glucose uptake in T24 cells and lactate uptake in fibroblasts. G) Changes in glucose and lactate levels in the cell supernatant, reflecting the rates of glucose consumption and lactate production in T24 cells with different pre-treatments or transfections, with the medium group serving as the control. H) Western blot analysis of CXCL14 and α-SMA expression in NFs under different treatments to evaluate fibroblast activation. I) Representative immunofluorescence images showing α-SMA and vimentin expression in NFs cocultured with CAFs or treated as specified. Scale bar, 10 μm. Data were presented as the means ± SDs, and experiments were performed at least three times. *P* values based on unpaired Student’s *t* test or one-way ANOVA followed by Tukey’s test; *P < 0.05 and **P < 0.01.

## Data Availability

No datasets were generated or analysed during the current study.
