# Supplementary material for: Cancer-associated fibroblast derived CXCL14 drives cisplatin chemoresistance by enhancing nucleotide excision repair in bladder cancer
Source: J Exp Clin Cancer Res. 2025 Sep 2;44:265. doi: 10.1186/s13046-025-03487-4 (PMC12406535; doi:10.1186/s13046-025-03487-4)
Supplement: Supplementary file 1 — Supplementary Material 1. Figure S1 A), B) Representative images illustrated E-cadherin and Vimentin expression in bladder cancer and adjacent tissues. Scale bar, indicates 100 μm for the 100 ×, and 20 μm for the 40 ×. C) Kaplan–Meier analysis was conducted to evaluate recurrence-free survival in bladder cancer patients undergoing chemotherapy. D), E) Kaplan–Meier analysis was conducted to evaluate overall survival and recurrence-free survival in two groups of bladder cancer patients stratified by CAF scores. F), G) Kaplan–Meier analysis was performed to evaluate cancer-specific survival (CSS) in two groups of bladder cancer patients stratified by CAF scores from two independent cohorts: 93 patients in the GSE31684 dataset and 424 patients in the GSE32894 dataset, respectively. H) Representative flow cytometry plots (left) demonstrating fibroblast event populations in primary extracted fibroblast cells. I), J) After 48 h treatment of cisplatin with different concentration, viabilities of T24 or UM-UC-3 cell with different conditional medium treatment and cisplatin were measured by CCK8 assay. K) Tumor growth curve of T24 cells alone or co-injected with NFs (T24-NFs) or CAFs (T24-CAFs). Tumor-bearing mice were intraperitoneally administered with cisplatin or PBS twice a week since the average tumor volume reached approximately 200 mm^3 (indicated by arrows). Categorical variables were compared through chi-squared test. Figure S2 A), B) The expression of MRP2 in T24 and UM-UC-3 cells with different conditional medium treatment were detected by Western blot. C), D) After 48 h treatment of cisplatin with different concentration, T24 or UM-UC-3 cell viabilities after cultured in different CM were measured by CCK8 assay. E), G) RT-qPCR analysis detecting MRP2 expression after different shRNAs transfection in T24 and UM-UC-3 cells. F), H) Western blot analysis assessed the translational expression of MRP2 in T24 and UM-UC-3 cells. I), J) After 48 h cisplatin treatment, via [file 13046_2025_3487_MOESM1_ESM.docx]

**Figure S1**


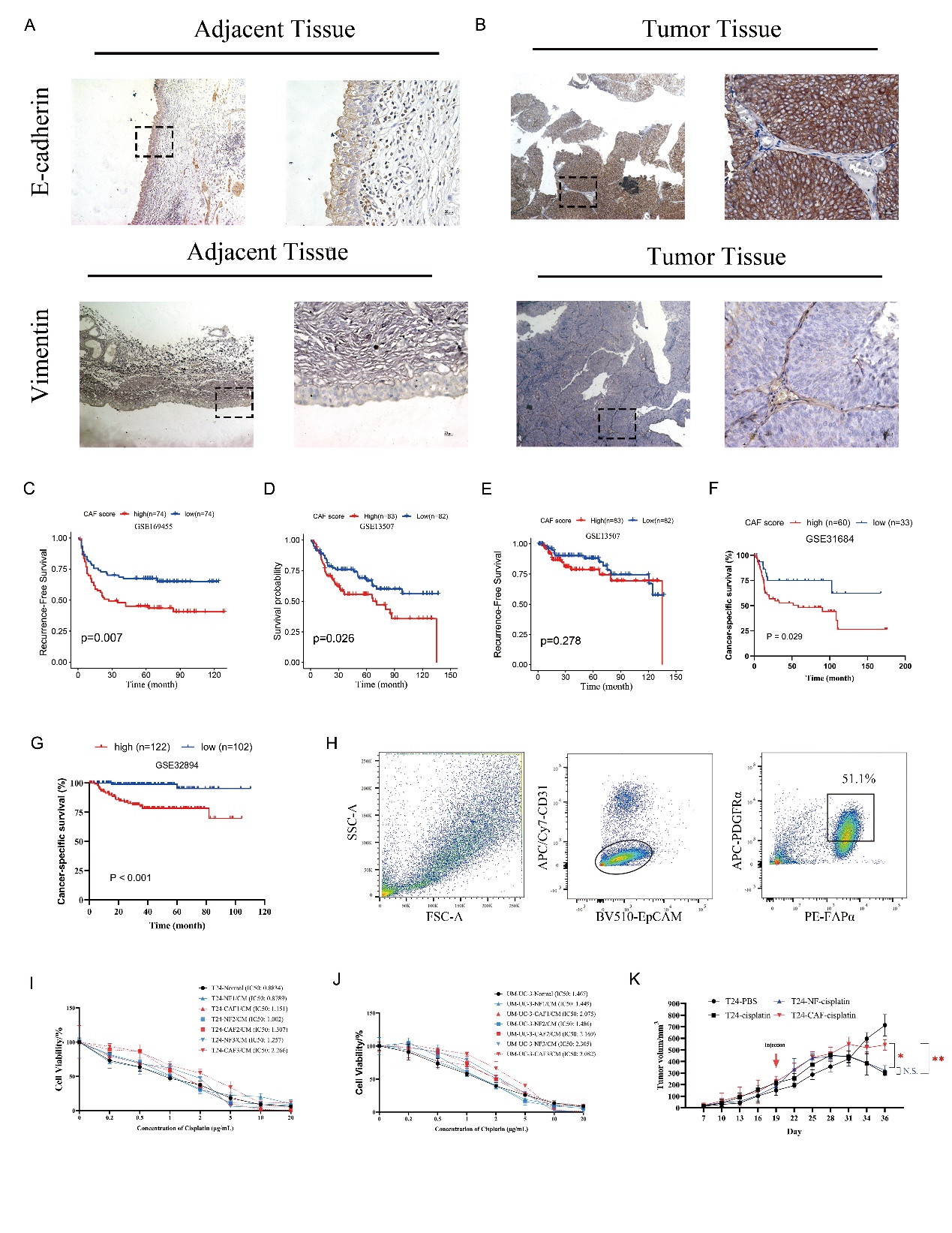


**A), B)** Representative images illustrated E-cadherin and Vimentin expression in bladder cancer and adjacent tissues. Scale bar, indicates 100 μm for the 100×, and 20 μm for the 40×. **C)** Kaplan-Meier analysis was conducted to evaluate recurrence-free survival in bladder cancer patients undergoing chemotherapy. **D), E)** Kaplan-Meier analysis was conducted to evaluate overall survival and recurrence-free survival in two groups of bladder cancer patients stratified by CAF scores. **F), G)** Kaplan-Meier analysis was performed to evaluate cancer-specific survival (CSS) in two groups of bladder cancer patients stratified by CAF scores from two independent cohorts: 93 patients in the GSE31684 dataset and 424 patients in the GSE32894 dataset, respectively. **H)** Representative flow cytometry plots (left) demonstrating fibroblast event populations in primary extracted fibroblast cells. **I), J)** After 48h treatment of cisplatin with different concentration, viabilities of T24 or UM-UC-3 cell with different conditional medium treatment and cisplatin were measured by CCK8 assay. **K)** Tumor growth curve of T24 cells alone or co-injected with NFs (T24-NFs) or CAFs (T24-CAFs). Tumor-bearing mice were intraperitoneally administered with cisplatin or PBS twice a week since the average tumor volume reached approximately 200 mm^3 (indicated by arrows). Categorical variables were compared through chi-squared test.

**Figure S2**


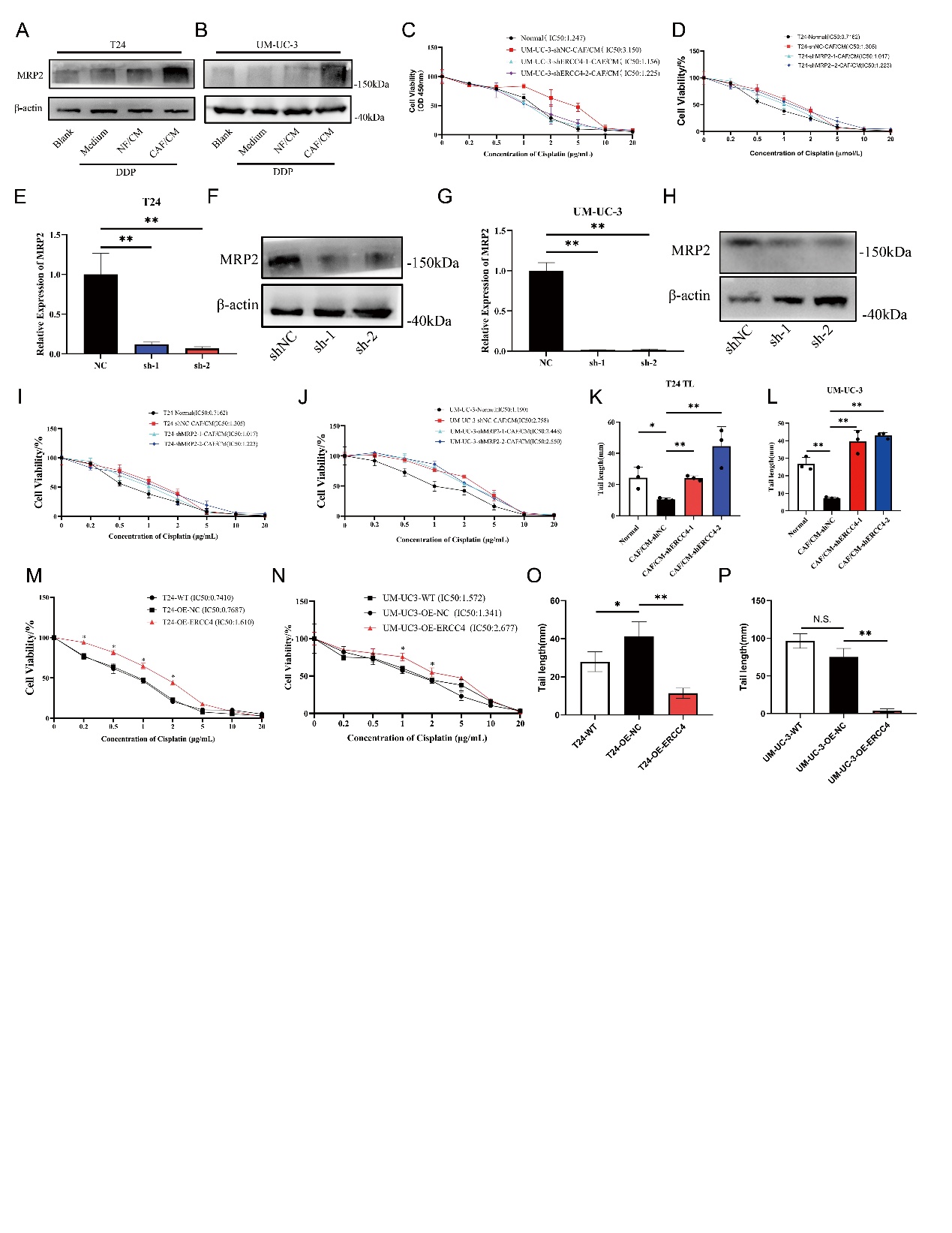


**A), B)** The expression of MRP2 in T24 and UM-UC-3 cells with different conditional medium treatment were detected by Western blot. **C), D)** After 48h treatment of cisplatin with different concentration, T24 or UM-UC-3 cell viabilities after cultured in different CM were measured by CCK8 assay. **E), G)** RT-qPCR analysis detecting MRP2 expression after different shRNAs transfection in T24 and UM-UC-3 cells. **F), H)** Western blot analysis assessed the translational expression of MRP2 in T24 and UM-UC-3 cells. **I), J)** After 48 h cisplatin treatment, viabilities of T24 or UM-UC-3 cells with different CM treatment were measured by CCK8 assay. **K), L)** Tail length was calculated using CASP software to evaluate the DNA damage levels in different groups of T24 and UM-UC-3 cells. **M), N)** After 48h cisplatin treatment, viabilities of T24 or UM-UC-3 cells with different lentivirus transfections were measured by CCK8 assay. **O), P)** Tail length was calculated using CASP software to evaluate the DNA damage levels in different groups of T24 and UM-UC-3 cells under treatment of cisplatin. P values based on unpaired Student’s *t* test or one-way ANOVA followed by Tukey’s test; *P < 0.05 and **P < 0.01.

**Figure S3**
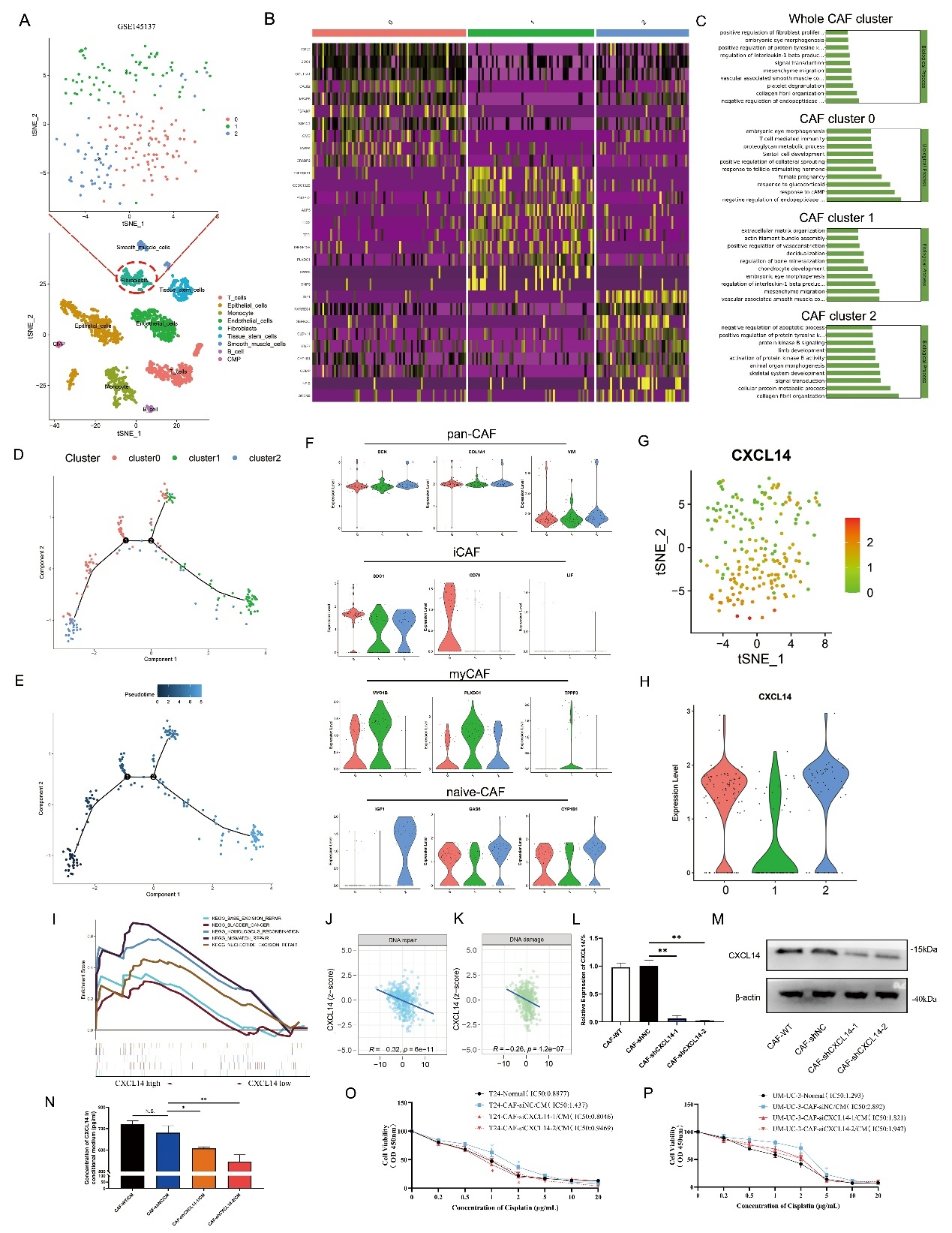


**A)** TSNE dimensionality reduction of all cells showing CAFs in chemoresistant bladder cancer samples, and re-clustering of CAFs identified three subpopulations. **B)** Heatmap demonstrated marker genes for each cluster. **C)** GO enrichment analysis demonstrated the enriched terms by feature genes of different subclusters. **D), E)** Pseudotime analysis demonstrated that cluster 2 was projected onto the root, cluster 0 was projected onto the trunk while cluster 1 were projected onto two branches, one as single and another differentiated with part of cluster 0. **F)** Representative images illustrated feature genes expression level in each cluster. **G), H)** CXCL14 exhibited high expression levels in both cluster 0 and 2. **I)** GSEA plots illustrating KEGG terms enriched in CXCL14 high-expression group, of particular significance in DDR-related pathways including “bladder cancer”, “base excision repair”, “mismatch repair”, “nucleotide excision repair” and “homologous recombination”. **J), K)** Pearson regression analysis of the TCGA data to access the correlation between CXCL14 expression and "Cell cycle" and "DNA damage" pathways. **L)** RT-qPCR analyzed the transcriptional expression of CXCL14 after transfection with different shRNAs in CAFs, with ACTB as the loading control. **M)** Western blot analysis assessing the translational expression of CXCL14 in CAFs transfected with different shRNAs. **N)** ELISA measurement of CXCL14 concentration in the CM of CAFs transfected with different shRNAs. **O), P)** After 48h treatment of cisplatin with different concentration, T24 or UM-UC-3 cell viabilities after cultured in different CM were measured by CCK8 assay. Data were presented as the means ± SDs, and experiments were performed at least three times. P values based on unpaired Student’s *t* test or one-way ANOVA followed by Tukey’s test; *P < 0.05 and **P < 0.01. Categorical variables were compared through chi-squared test, and Pearson correlation coefficient was used for correlation analysis.

**Figure S4**


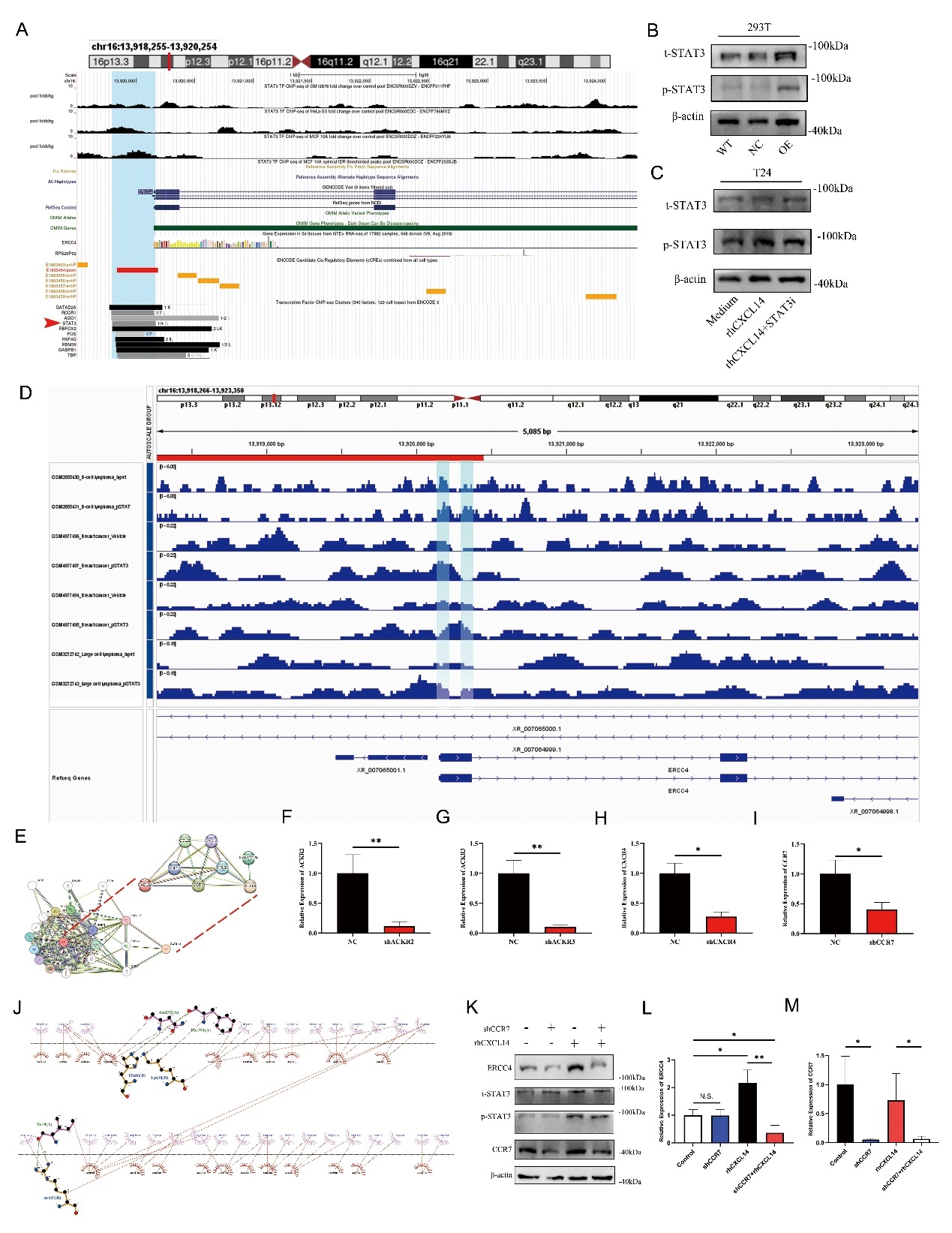


**A)** STAT3 binding peaks within the promoter region of ERCC4 were visualized using the ENCODE database (www.encodeproject.org) in GM12878, MCF-10A, and HeLa-S3 cells (highlighted in blue and indicated by red arrows). **B)** STAT3 overexpression in 293T cells was confirmed by Western blot. **C)** Activation of STAT3 by rhCXCL14 and its inhibition by a STAT3 inhibitor (STAT3i) in T24 cells were demonstrated via Western blot analysis. **D)** IGV exhibiting seven CHIP-seq data sourced from three type of cancer including B cell lymphoma, T cell lymphoma and breast cancer, demonstrated the enrichment of STAT3 in the promoter of ERCC4 (highlighted in blue).­­­ **E)** The protein interaction network was predicted by STRING (<https://cn.string-db.org/>). **F-I)** RT-qPCR analysis detecting efficiency of different shRNA’s inhibition on andidate receptors for CXCL14 including ACKR2, ACKR3, CXCR4, and CCR7 in T24 cells. **J)** The specific binding sites of CCR7 and CXCL14 were predicted using AutoDock Vina(<https://vina.scripps.edu/>). **K)** Western blot analysis of ERCC4, CCR7, STAT3, and phosphorylated STAT3 expression in T24 cells treated with recombinant human CXCL14, with or without CCR7 knockdown. **L), M)** RT-qPCR analysis detected ERCC4 and CCR7 expression in T24 cells under different treatment. Data were presented as the means ± SDs, and experiments were performed at least three times. P values based on unpaired Student’s *t* test or one-way ANOVA followed by Tukey’s test; *P < 0.05 and **P < 0.01. Categorical variables were compared through chi-squared test, and Pearson correlation coefficient was used for correlation analysis.

**Figure S5**


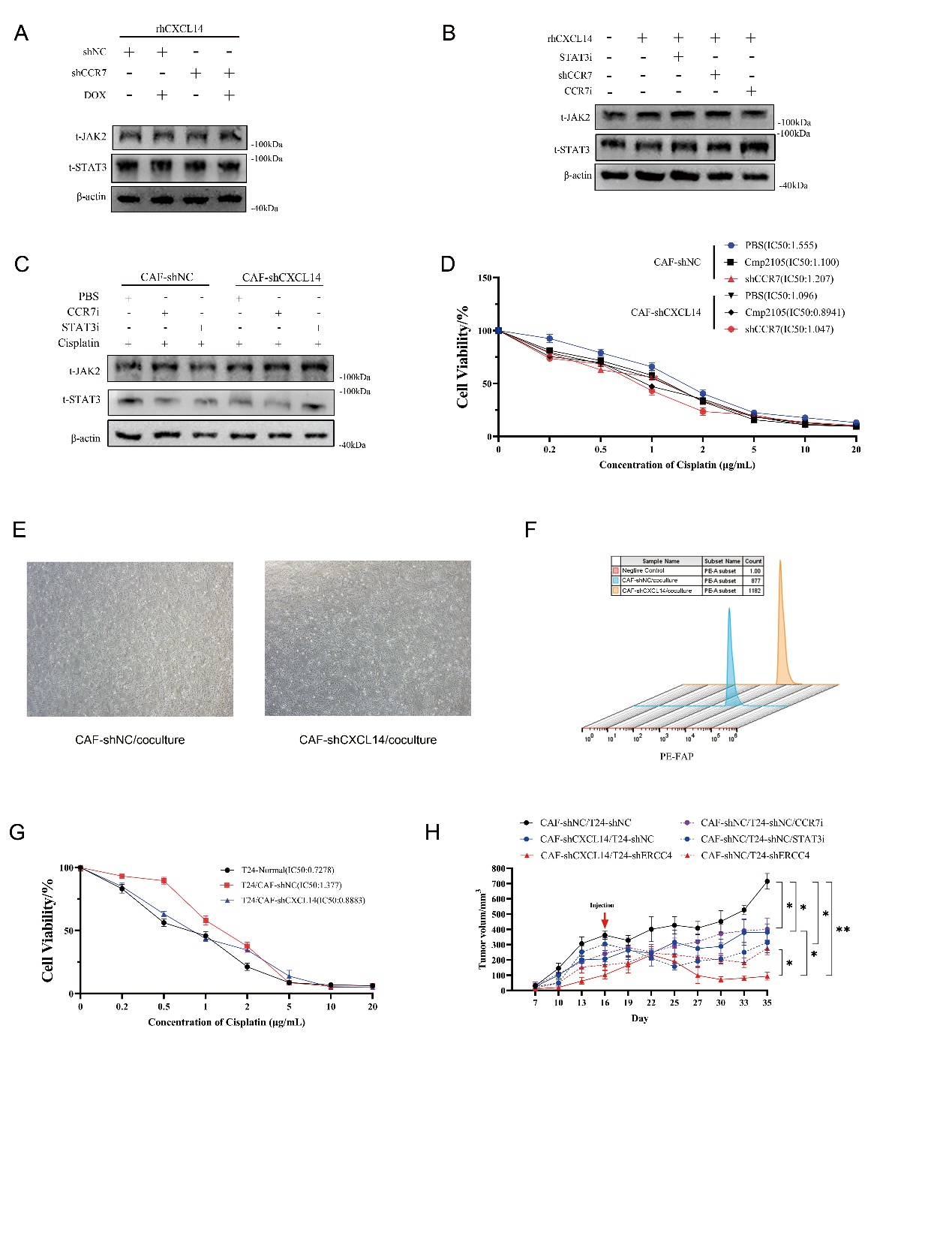


**A)** Western blotting was conducted to analyze the expression of STAT3 and JAK2 in T24 cells with or without inducible knockdown of CCR7. **B)** Western blot analysis examining total STAT3 and JAK2 expression in T24 cells under different treatment of inhibitors or knockdown of CCR7. **C)** Western blot analysis examining STAT3 and JAK2 expression in T24 cells of different groups in the presence of 1 μg/mL cisplatin. **D)** After 48 h of cisplatin treatment, T24 cell viability in different groups was assessed using the CCK-8 assay. **E)** Representative images of T24 cells co-cultured with CAFs transfected with different lentiviruses. **F)** Following co-culture, FAP-positive fibroblasts were sorted using flow cytometry, while T24 cells without FAP expression were retained for subsequent analyses. **G) A**fter 48 h of cisplatin treatment, the viability of sorted T24 cells co-cultured with different CAFs was assessed using the CCK-8 assay. **H)** Tumor growth curves of T24 cells with different shRNA transfections coinjected with CAFs deficient in CXCL14 expression or negative control. Tumor-bearing mice were intraperitoneally administered cisplatin along with medium or inhibitors twice a week, starting when the average tumor volume reached approximately 200 mm^3 (indicated by arrows). Data were presented as the means ± SDs, and experiments were performed at least three times. P values based on unpaired Student’s *t* test or one-way ANOVA followed by Tukey’s test (*P<0.05 and **P<0.01).

**Figure S6**


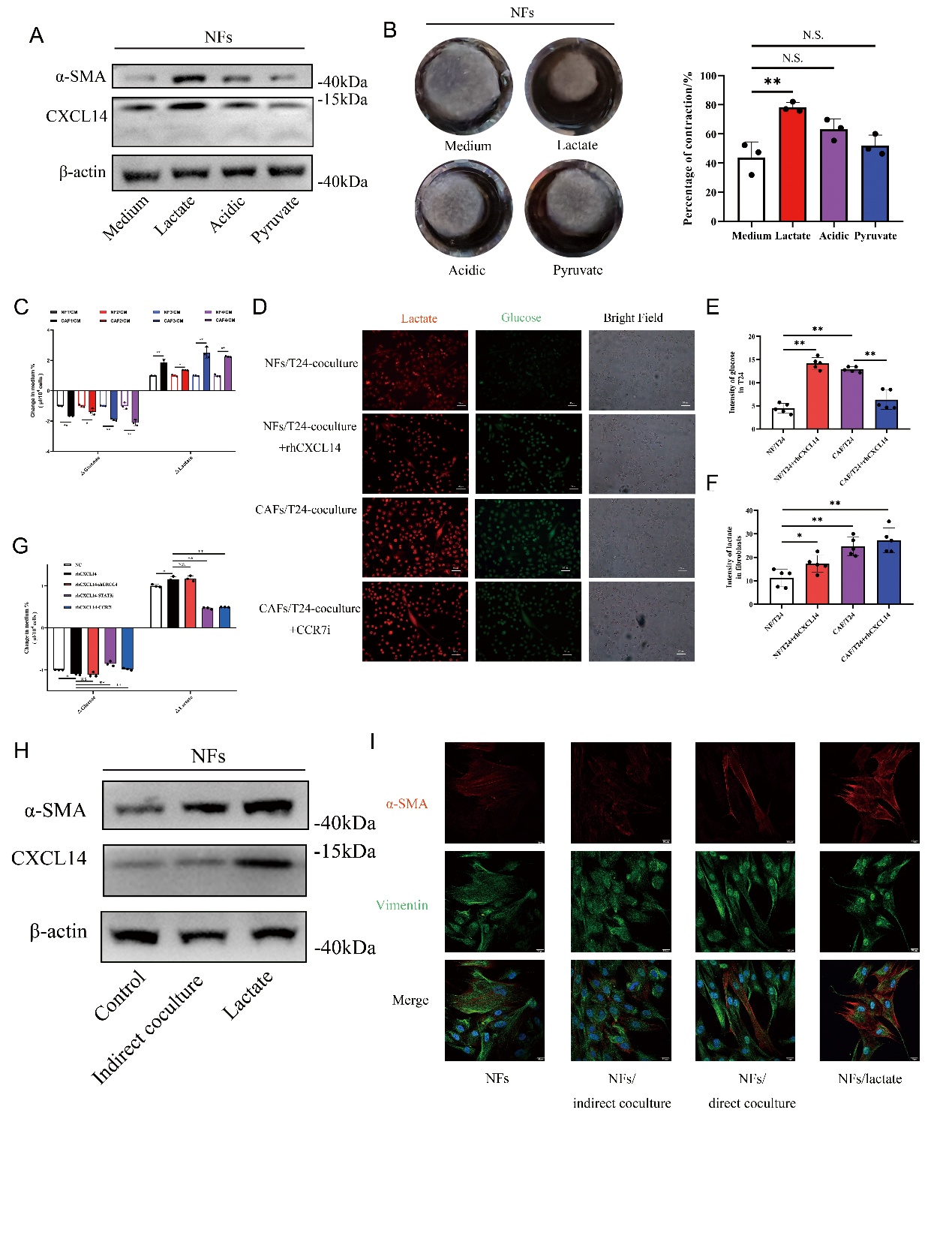


**A)** Western blot analysis of CXCL14 and α-SMA expression in NFs exposed to different metabolites or simulated acidic microenvironments **B)** Collagen contraction assay was assessed for the contraction ability of NFs treated as indicated, and results were quantified using ImageJ software. **C)** Changes in glucose and lactate levels in the cell supernatant, reflecting the rates of glucose consumption and lactate production in T24 cells treated with different CAFs and paired NFs, with each group compared to its corresponding NF. **D)** Representative images illustrating glucose uptake (green fluorescence) and lactate uptake (red fluorescence) in T24 cells cocultured with NFs or CAFs and stimulated with various drugs. White light images were included to observe cell morphology and distinguish between cell types. Scale bar, 50 μm. **E), F)** Immunofluorescence quantification of glucose uptake in T24 cells and lactate uptake in fibroblasts. **G)** Changes in glucose and lactate levels in the cell supernatant, reflecting the rates of glucose consumption and lactate production in T24 cells with different pre-treatments or transfections, with the medium group serving as the control. **H)** Western blot analysis of CXCL14 and α-SMA expression in NFs under different treatments to evaluate fibroblast activation. **I)** Representative immunofluorescence images showing α-SMA and vimentin expression in NFs cocultured with CAFs or treated as specified. Scale bar, 10 μm. Data were presented as the means ± SDs, and experiments were performed at least three times. P values based on unpaired Student’s *t* test or one-way ANOVA followed by Tukey’s test; *P < 0.05 and **P < 0.01.
